# Birth outcomes for women with pre-existing mental health problems: a systematic review and meta-analysis

**DOI:** 10.1136/bmjopen-2025-106566

**Published:** 2026-05-29

**Authors:** Jenny Gong, Ian Henderson, Rosie Lynch, Zoe Daskalopoulou, Nia Roberts, Gracia Fellmeth, Sian Harrison, Maria A Quigley, Fiona Alderdice

**Affiliations:** 1 National Perinatal Epidemiology Unit, University of Oxford Nuffield Department of Population Health, Oxford, UK; 2 Gove District Hospital, Nhulunbuy, Northern Territory, Australia; 3 Bodleian Health Care Libraries, Knowledge Centre ORC Research Building, University of Oxford, Oxford, UK

**Keywords:** Systematic Review, Meta-Analysis, MENTAL HEALTH, Pregnancy

## Abstract

**Abstract:**

**Objectives:**

To synthesise evidence on the association between any diagnosed or self-reported mental health problems prior to pregnancy (pre-existing mental health problems) and birth outcomes including preterm birth (PTB), low birth weight (LBW), small for gestational age (SGA), neonatal unit (NNU) admission and mode of birth (instrumental birth, planned or unplanned caesarean section).

**Methods:**

Systematic searches were conducted in MEDLINE, CINAHL, Embase and PsycINFO in December 2024 for studies examining the association between any pre-existing mental health problems and PTB, LBW, SGA, NNU admission and mode of birth. Only articles published in English were included with no restriction on year of publication. Two reviewers independently screened studies and extracted data. Study quality was assessed using the Newcastle-Ottawa Scale and Joanna Briggs Institute checklists. Random-effects meta-analyses were conducted to pool crude and adjusted ORs (aORs) and risk ratios (aRR) with 95% CIs. ORs and RRs were analysed separately. Between-study heterogeneity was quantified using the I^2^ statistic.

**Results:**

Of 15 467 records screened, 33 studies met the inclusion criteria. Women with any pre-existing mental health problems had higher odds and risks of adverse birth outcomes, including PTB (aOR 1.41, 95% CI 1.27 to 1.56) (aRR 1.36, 95% CI 1.21 to 1.51), LBW (aOR 1.28, 95% CI 1.22 to 1.33) (aRR 1.32, 95% CI 1.04 to 1.68), SGA (aOR 1.27, 95% CI 1.07 to 1.51) (aRR 1.34, 95% CI 1.19 to 1.51) and NNU admission (aOR 1.44, 95% CI 1.19 to 1.74). Adjusted estimates were based on multivariable models that commonly controlled for maternal age, parity and socio-demographic factors. No consistent associations were observed between pre-existing mental health problems and mode of birth.

**Conclusions:**

Pre-existing mental health problems were associated with increased risks and odds of several adverse birth outcomes. These findings highlight the importance of early identification and targeted support for women with mental health problems before pregnancy to strengthen preconception and maternity care planning.

**PROSPERO registration number:**

CRD42023485834.

**STRENGTHS AND LIMITATIONS OF THIS STUDY**

- This systematic review employed a robust and comprehensive search strategy, supplemented with manual hand-searching, to maximise the likelihood of capturing all relevant studies.
- A clear and consistent definition of pre-existing mental health problems was applied to ensure preconception onset and minimise potential misclassification or dilution of results due to antenatal mental health problems.
- Crude and adjusted ORs and risk ratios were pooled separately to examine the effect of adjustment for confounders.
- Only studies published in English were included, therefore relevant studies published in other languages may have been excluded.
- Despite a consistent eligibility definition, included studies varied in how pre-existing mental health problems were operationalised and measured, as well as in the timing of exposure assessment, types of mental health problems examined and covariates adjusted for. These differences may have contributed to heterogeneity and residual confounding.

## Introduction

 Mental health problems affect approximately 10–16% of pregnant women and up to 20% of women after birth.^1^ However, many mental health problems have their onset before pregnancy and may persist or recur over the reproductive life course. A history of mental health problems prior to pregnancy (pre-existing mental health problems) is a well-established risk factor for poor mental health during pregnancy and the postpartum period.^1 2^ Despite this, the implications of pre-existing mental health problems for subsequent birth outcomes remain poorly understood, limiting the evidence available to inform preconception risk assessment and care.

Existing literature has consistently shown that mental health problems during pregnancy are associated with adverse outcomes such as preterm birth (PTB), low birth weight (LBW) and small-for-gestational-age (SGA).^3^,^6^ However, many studies do not distinguish between mental health problems that predate pregnancy and those with onset during pregnancy, making it difficult to disentangle the role of pre-existing mental health problems from antenatal exposure. This review focuses specifically on pre-existing mental health problems to avoid potential misclassification with antenatal mental health problems.

PTB, LBW and SGA are clinically significant outcomes associated with increased risks of neonatal morbidity and mortality, as well as adverse health outcomes across the life course.^7^,^11^ Infants born preterm are at increased risk of immediate complications such as respiratory distress syndrome and infection, while infants born with LBW or SGA experience higher rates of neonatal mortality and long-term metabolic and cardiovascular morbidity.^7^,^11^ The economic burden of these outcomes is substantial, encompassing both short-term neonatal unit (NNU) costs and longer-term health monitoring and healthcare utilisation expenses.^12^

While the effects of antenatal mental health problems and their treatments have been widely studied, there is a lack of systematic evidence on the effects of pre-existing mental health problems, despite it being acknowledged as a major risk factor for poor perinatal mental health^13^ as well as a predictor of adverse outcomes such as pregnancy loss.^14^ One meta-analysis examined pre-existing mental health problems, but researchers focused solely on stillbirth and infant mortality.^15^ To our knowledge, Montagnoli *et al*^16^ conducted the only review examining both pre-existing and antenatal mental health problems in relation to obstetrical and reproductive outcomes. However, this scoping review was limited by its search strategy which did not capture all relevant literature. Consequently, the extent to which pre-existing mental health problems are associated with a wider range of birth outcomes remains unclear.

This systematic review and meta-analysis aims to address this key gap in the literature by comprehensively examining the association between pre-existing mental health problems and a broad range of birth outcomes including PTB, LBW, SGA, NNU admission and mode of birth. These birth outcomes were selected due to their clinical significance and established links to adverse maternal and infant outcomes.^3 4 17 18^

## Methods

### Search strategy

A systematic literature search was conducted in the following databases: MEDLINE, Embase, CINAHL and PsycINFO. In collaboration with an experienced university librarian, a comprehensive search strategy was developed and tailored to each database. The final search took place on 24 November 2023, with an updated search conducted on 12 December 2024. The full search strategies used for each database are presented in online supplemental tables S1a–d. Searches were limited to studies published in English, with no restriction on year of publication. Grey literature was excluded. The reference lists and citations of eligible articles were manually screened to identify additional eligible studies. This review was registered with PROSPERO (CRD42023485834).

### Inclusion and exclusion criteria

All observational studies examining the association between pre-existing mental health problems and birth outcomes were eligible. Pre-existing mental health problems were defined as any self-reported or clinically diagnosed mental health problem at any point prior to pregnancy. The comparator was birth outcomes for women without pre-existing mental health problems. Birth outcomes of interest included PTB, LBW, SGA, NNU admission and mode of birth. Within mode of birth, we examined instrumental birth and caesarean section, compared with normal vaginal birth. Where data permitted, caesarean section was further categorised as planned or unplanned caesarean section to allow more detailed analyses. Only studies published in English and conducted in high-income countries as classified by the World Bank (2023) were included.

No restrictions were placed on type of mental health problem. However, studies examining substance use disorders exclusively were excluded, as these conditions may have more direct and greater physiological effects on birth outcomes. Women with substance use disorders may also face additional risk factors, potentially confounding the association between pre-existing mental health problems and birth outcomes.

### Study selection and data extraction

All citations were imported into Covidence and duplicates were removed. Two reviewers independently screened titles, abstracts and full-text articles and independently extracted data from included studies using a standardised, pre-piloted data extraction form. Discrepancies were resolved through consensus, or discussion with other team members when necessary.

The information extracted included study characteristics, the definition and type of pre-existing mental health problems, method of assessing mental health problems and birth outcomes examined. Crude and adjusted estimates such as ORs, relative risks (RR) or HRs, along with 95% CIs and confounders were also extracted. For studies that did not report effect estimates, crude measures were manually calculated where possible.

### Study quality and risk of bias

We used the Joanna Briggs Institute (JBI) Critical Appraisal Checklist^19^ for cross-sectional studies and adapted the Newcastle-Ottawa Quality Assessment Scale (NOS)^20^ for cohort and case–control studies to assess study quality and risk of bias.

The JBI Critical Appraisal Checklist includes eight questions, scored using ‘yes’, ‘no’, ‘unclear’ or ‘not applicable’. Studies are assessed based on the following domains: (1) inclusion and exclusion criteria, (2) sampling, (3) exposure measurement, (4) method of ascertaining disease, (5) identification of confounders, (6) adjustment of confounders, (7) measurement of outcomes and (8) appropriate statistical analysis methodology.^19^ Additionally, the NOS for cohort studies includes eight questions under three overarching domains: (1) selection or sample representativeness, (2) comparability of exposed and unexposed groups and (3) assessment of study outcomes.^20^

Cohort studies were not given numerical scores. Instead, each question on the scale was scored using ‘yes’, ‘no’, ‘unclear’ or ‘not applicable’, similar to the JBI Critical Appraisal Checklist. For both appraisal tools, studies that scored ‘yes’ in all domains were classified as ‘higher quality’. Two reviewers independently performed the quality and risk of bias assessments, and disagreements were resolved through consensus.

### Data analysis

Random-effects meta-analyses were used to estimate the pooled effect sizes and 95% CIs for associations between any pre-existing mental health problems and each birth outcome. Crude and adjusted ORs and RRs were pooled separately to retain the original effect measures reported by studies and to avoid potential bias introduced by transforming estimates. A random effects model was selected to account for anticipated clinical and methodological heterogeneity across studies, including differences in the types of mental health problems examined, exposure and outcome measurement and geographical setting. Between-study heterogeneity was examined using the I^2^ statistic, where I^2^ <50% was considered low, 50–75% moderate and >75% high heterogeneity.^21^ Potential publication bias was graphically examined using funnel plots and further quantified using Egger’s test, with p<0.05 indicating significant publication bias.^22^ Testing for publication bias was only conducted for analyses involving 10 or more studies to ensure sufficient power.^21^ All analyses were conducted using Stata V.17 (StataCorp, College Station, Texas, USA).

### Sensitivity analyses

Three sensitivity analyses were conducted to assess the robustness of findings by restricting studies based on: (1) study quality, (2) type of mental health problem and (3) type of comparison group. The first analysis included only higher quality studies that met all criteria on the JBI or NOS checklists. The second grouped studies by type of mental health problem based on the National Institute for Health and Care Excellence guidance, examining associations between each birth outcome and pre-existing common mental health problems,^23^ severe mental health problems^24^ and eating disorders separately. Common mental health problems included depression, anxiety, panic disorder, obsessive-compulsive disorder and post-traumatic stress disorder (PTSD),^23^ while severe mental health problems referred to bipolar disorder, psychosis and schizophrenia.^24^ The third sensitivity analysis addressed heterogeneity in comparison groups. Studies with a comparison group free from any pre-existing mental health comorbidities were classified as the ‘no comorbidity’ group. In contrast, studies that either reported comorbid mental health problems in the comparison group or adjusted for comorbidity in their analyses were categorised as ‘possible comorbidity’.

## Results

### Search results

**Figure 1:**
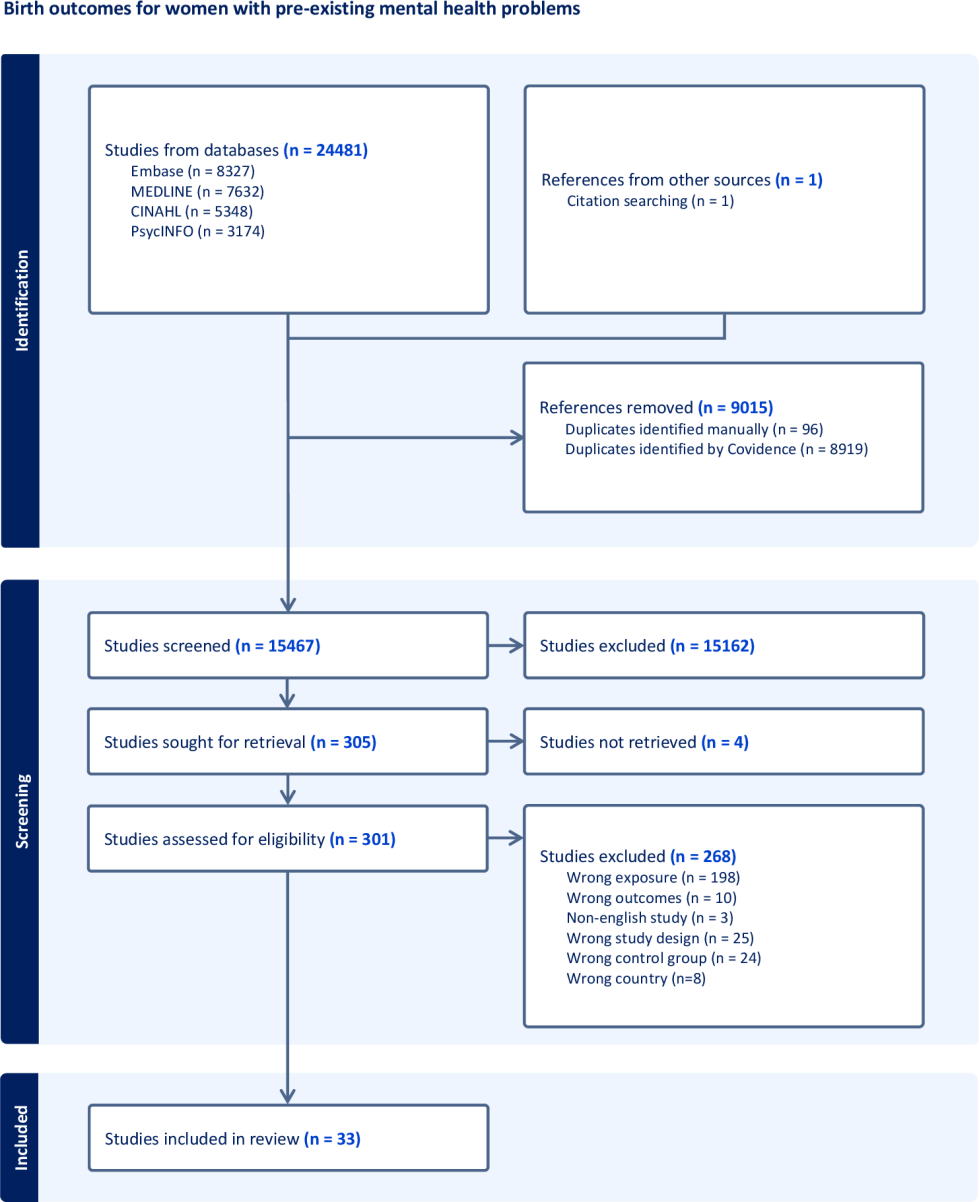
Preferred Reporting Items for Systematic Reviews and Meta-Analyses 2020 flow diagram for the systematic review and meta-analysis of pre-existing mental health problems and birth outcomes.^25^

Results are presented in PRISMA^25^ format in figure 1. A total of 24 481 studies were identified through database searching with one additional study identified through citation searching. After removing duplicates, 15 467 studies were screened by title and abstract and 301 studies were assessed in full-text review. Of these, 268 studies were excluded. The most common reason for exclusion was incorrect exposure (n=198), where timing of onset of mental health problems was unclear or the study examined antenatal mental health problems only. 13 authors were contacted to clarify the timing of onset of mental health problems but only one responded. Therefore, 33 studies were included in this systematic review.^26^,^58^

### Characteristics of included studies

The included studies are described in table 1. The sample size of included studies ranged from 116 to 2 134 945 births. The main pre-existing mental health problems examined included: depression symptoms or clinical depression (n=9),^35^,^3741 43 47 48 57 58^ anorexia nervosa (AN) (n=6),^27 29 30 34 44 55^ schizophrenia (n=2)^52 53^ and PTSD (n=1).^49^ In three studies, depression and/or anxiety were combined.^28 32 51^ One study examined affective disorders,^26^ which included diagnoses such as bipolar disorder, depressive disorder, recurrent depressive disorder, persistent mood affective disorder and unspecified or other mood affective disorders. Two studies looked at any eating disorders,^39 50^ which included AN, bulimia nervosa and Eating Disorders Not Otherwise Specified (EDNOS), while one study examined only EDNOS.^42^ Eight studies examined unspecified pre-existing mental health problems.^31 33 38 40 45 46 54 56^ The outcomes reported were: PTB (n=27),^26^,^3032^ SGA (n=18),^27^,^3037 39 40 42^ LBW (n=13),^2728 30 32^,^34 45 46 50 53 56^ combined SGA or intrauterine growth restriction (IUGR) (n=1),^31^ NNU admission (n=3),^28 41 57^ and mode of birth. Within mode of birth, studies reported caesarean section (n=13)^29 30 32 34 39 42 45 48 52 53 55 57 58^ and instrumental birth (n=8).^32 34 39 42 48 53 55 57^ In some studies, caesarean section was separated into planned caesarean section (n=3)^32 39 57^ and unplanned caesarean section (n=3).^32 39 57^ The majority of studies were conducted in the USA (n=8)^31 35 36 38 41 47 49 56^ and Canada (n=4),^27 43 52 53^ as well as the Scandinavian countries Denmark (n=5),^30 37 44 45 50^ Sweden (n=3)^39 42 57^ and Norway (n=2).^29 55^ Three studies^34 40 46^ were conducted in the UK, two^28 32^ in Italy and two^33 51^ in Australia. One study was conducted in each of the following countries: Spain,^26^ Finland,^48^ China (Hong Kong)^54^ and Israel.^58^ The vast majority of the studies were cohort studies (n=28)^26^,^3032^ and five studies were cross-sectional.^31 41 46 48 56^ Aside from maternal age, which was adjusted for in the majority of the studies, the included studies varied considerably in terms of the other covariates they adjusted for (see online supplemental table S2).

**Table 1:** Study characteristics

Author	Country (region)	Study design	Sample size	Data source	Main mental health problems examined (n)*	Definition of pre-existing mental health	Birth outcomes
Aliaga *et al*^*26*^	Spain (Catalonia)	Cohort	102 086 pregnancies	Enhancement of Research in Primary Care (SIDIAP) databases	Affective disorders (n=1631 women)	AD presenting before pregnancy	PTB
Ante *et al*^27^	Canada (Quebec)	Cohort	2 134 945 singleton pregnancies	Hospital clientele registry	AN (n=1842 women)	Hospitalisation for AN before pregnancy	PTB, LBW, SGA
Bua *et al*^28^	Italy (national)	Cohort	6814 mother–child pairs	Nascita e INFanzia: gli Effetti dell’Ambiente	Composite depression or anxiety (n=525 women)	Self-reported mental disorder diagnosed by a clinician before pregnancy	PTB, LBW, SGA, NNU admission
Bulik *et al*†^29^	Norway (national)	Cohort	35 929 singleton pregnancies	Norwegian Mother and Child Cohort Study (MoBa)	AN (n=35 women)	Individuals who met AN criteria 6 months before pregnancy	CS, PTB, SGA
Chatwin *et al*^30^	Denmark (national)	Cohort	1 517 839 singletons	Danish Medical Birth Registry (linked)	AN (n=4957 women)	AN diagnosed more than 2 years before pregnancy	CS, PTB, LBW, SGA
Ciesielski *et al*‡^31^	USA (Providence Rhode Island)	Cross-sectional	490 deliveries	Medical records from the Women and Infants Hospital in Providence	Depression/anxiety/obsessive compulsive disorder (n=74 women)	History of psychiatric diagnosis before pregnancy	SGA or IUGR (intrauterine growth restriction)
Corti *et al*§^32^	Italy (Milan)	Case–control	149 women	Medical records from Sacco Hospital, Milan	Depression/anxiety (n=42 women)	Diagnosis and/or treatment before pregnancy	Instrumental birth, planned CS, unplanned CS, CS, PTB, LBW
Dadi *et al*^33^	Australia (Northern Territory)	Cohort	72 518 births	Northern Territory Inpatient Activity Collection Dataset	One or more of any mental health problems	Hospitalisation for mental health in the 5 years before pregnancy (n=425 women)	PTB, LBW
Eagles *et al*^34^	Scotland (Northeast region)	Cohort	916 women	Aberdeen Maternal and Neonatal Databank	AN (n=134 women, 230 births)	AN diagnosed before pregnancy	Instrumental birth, CS, PTB, LBW
Gavin *et al*^35^	USA (Michigan)	Cohort	3030 women	Michigan-based Pregnancy Outcomes and Community Health study	Depression (n=624 women)	Self-reported maternal history of depression	PTB
Haas *et al*^36^	USA (San Francisco)	Cohort	1619 singleton deliveries	Project WISH (Women and Infants Starting Healthy)	Depression (n=184 women)	Self-report during telephone survey	PTB
Jensen *et al*^37^	Denmark (national)	Cohort	673 853 singleton deliveries	Danish Medical Birth Register	Depression (n=3287 women)	Diagnosis of depression before pregnancy	SGA
Kang-Yi *et al*^38^	USA (Pennsylvania)	Cohort	9930 women	Pennsylvania Medicaid claims data	Any (n=4965 women)	Diagnosis or treatment before pregnancy	PTB
Kouba *et al*^39^	Sweden (Stockholm)	Cohort	116 women	Women recruited from 13 prenatal clinics	Eating disorders (n=49)	History of pre-existing eating disorder symptoms obtained by trained midwife	Instrumental birth, planned CS, unplanned CS, CS, PTB, SGA
Langham *et al*^40^	England (national)	Cohort	2 081 043 singleton deliveries	Hospital Episode Statistics	Any (n=1 51 770)	Contact with specialist mental healthcare in the 7 years before pregnancy onset	PTB, SGA
Latendresse *et al*^41^	USA (Utah)	Cross-sectional	4682 singleton births	Pregnancy Risk Assessment Monitoring System database	Depression	Self-reported mental health problem before pregnancy	NNU admission
Mantel *et al*^42^	Sweden (national)	Cohort	1 295 550 births	Swedish Medical Birth Register	AN (n=4938), bulimia nervosa (n=2629), other (n=6987)	Diagnoses before pregnancy	Instrumental birth, CS, PTB, SGA
Mei-Dan *et al*^43^	Canada (Ontario)	Cohort	1 032 831 obstetrical deliveries	Population-level health administrative data	MDD (n=3724 unique women with 4487 deliveries)	Hospitalisation for mental health problem 5 years before conception	PTB, SGA
Micali *et al*^44^	Denmark (national)	Cohort	83 826 women	Danish Medical Birth Register (linked)	AN (n=1262)	Self-reported history of AN before pregnancy	SGA
Momen *et al*^45^	Denmark (national)	Cohort	1 132 757 singleton pregnancies	Danish Medical Birth Register (linked)	Any mental health disorder (n=48 646)	Any mental disorder diagnosed more than 2 years prior to conception	CS, PTB, LBW, SGA
Mongan *et al*^46^	Northern Ireland, UK	Cross-sectional	140 569 singleton pregnancies	Northern Ireland Maternity System	Composite (n=26 547 singleton pregnancies)	Self-reported pre-existing mental disorders during assessment with trained midwife	PTB, LBW
Phillips *et al*^47^	USA (national)	Cohort	2627 singleton births	The Black Women’s Health Study	Depression (n=290 births, 274 mothers)	CES-D¶ score equal or greater than 23 before pregnancy	PTB
Räisänen *et al*^48^	Finland (national)	Cross-sectional	511 938 women	Finnish medical birth register	Depression (n=16 712 women)	Women with history of depression before pregnancy	Instrumental birth, CS
Shaw *et al*^49^	USA (national)	Cohort	14 047 women and 16 334 deliveries	Veterans Health Administration Database	PTSD (n=1128)	PTSD more than 365 days before the day of delivery	PTB
Sollid *et al*^50^	Denmark (national)	Cohort	2056 singleton deliveries	Danish Database for Psychiatric Epidemiological Research	Eating disorders (n=302 women, 504 births)	Hospitalisation for ED before pregnancy	PTB, LBW, SGA
Spry *et al*^51^	Australia (Victoria)	Cohort	398 women and 609 infants	The Victorian Intergenerational Health Cohort Study	Common mental health problems (n=162 women)	Diagnosis before pregnancy	PTB, SGA
Vigod *et al*^52^	Canada (Ontario)	Cohort (2006–2011)	732 264 deliveries to 5 74 495 women	Linkage across multiple clinical databases	Schizophrenia (n=4279 deliveries)	Hospitalisation or outpatient contacts prior to conception	CS, PTB, SGA
Vigod *et al*^53^	Canada (Ontario)	Cohort (2002–2011)	433 749 women	Linkage across multiple clinical databases	Schizophrenia (n=1628 deliveries)	Hospitalisation or service claims prior to conception	Instrumental birth, CS, PTB, SGA, LBW
Wang *et al*^54^	China (Hong Kong)	Cohort	411 251 mother–child pairs	Hong Kong Clinical Data Analysis and Reporting System	Those who used antipsychotics	Use of antipsychotics before pregnancy who discontinued receipt of treatment when pregnant	PTB, SGA
Watson *et al*^55^	Norway (national)	Cohort	65 995 women	Norwegian Mother and Child Cohort Study (MoBa)	AN (n=409 women)	AN in the 6 months before pregnancy	Instrumental birth, CS, PTB, SGA
Witt *et al*^56^	USA (national)	Cross-sectional	2108 women	Medical Expenditure Panel Survey	Poor mental health (n=143 women)	Self-reported a global mental health rating of ‘fair’ or ‘poor’ before pregnancy	LBW
Wolgast *et al*^57^	Sweden (national)	Cohort	262 329 women	Swedish medical birth register	MDD (n=5652 women)	Medication and diagnosis with an MDD within 12 months before pregnancy	Instrumental birth, planned CS, unplanned CS, CS, PTB, LBW, SGA, NNU admission
Yedid Sion *et al*^58^	Israel (Southern region)	Cohort	256 312 births	Medical records from Soroka University Medical Center	Depression (n=221 women)	Diagnosis of depression before pregnancy	CS, PTB, LBW

* The mental health problem shown reflects the primary exposure reported in each study; some studies also examined additional specific conditions. Where studies reported additional specific mental health conditions and corresponding effect estimates, these are detailed in online supplemental table S2.

† As Bulik *et al*^29^ and Watson *et al*^55^ used the same dataset, only one study was included per outcome in pooled analyses to avoid double counting study participants. Where two or more publications used the same underlying cohort or administrative dataset, only one paper per outcome was included in pooled analyses to avoid double-counting of participants. If multiple papers from the same dataset reported the same outcome, we prioritised the most recent study or the one reporting the most complete and/or adjusted effect estimates (eg, Watson *et al*^55^ was prioritised over Bulik *et al*^29^; Momen *et al*^45^ was prioritised over Micali *et al*^44^). A detailed description of potential overlaps in data sources, including how overlap was assessed and addressed for each pooled analysis, is provided in online supplemental 20.

‡ Ciesielski *et al*^31^ was not included in the meta-analysis because the study reported a combined outcome of small for gestational age (SGA) and intrauterine growth restriction (IUGR), which was not directly comparable with studies reporting SGA separately.

§ Corti *et al*^32^ was described by authors as a nested case control study, however, the study was conducted and reported as a cohort study.

¶ Center for Epidemiologic Studies Depression Scale.

AD, affective disorder; AN, anorexia nervosa; CS, caesarean section; ED, eating disorder; LBW, low birth weight; MDD, major depressive disorder; NNU, neonatal unit; PTB, preterm birth; PTSD, post-traumatic stress disorder.

### Methodological quality and risk of bias

Online supplemental table S3 describes the risk of bias for cohort and cross-sectional studies included in this review. Of the 28 cohort studies assessed using the adapted NOS,^26^,^3032^ 11 studies were categorised as higher quality as they scored ‘yes’ under all questions in every domain.^27 30 34 37 40 42 45 50 52 53 57^ Out of five cross-sectional studies assessed using the JBI Critical Appraisal Checklist,^31 41 46 48 56^ there was one study rated as higher quality.^46^

Across studies, the most common methodological limitations related to the representativeness of the exposed population and completeness of follow-up. These concerns were particularly evident in smaller or regionally recruited cohorts, such as the Veterans Health Administration cohort,^49^ which may not be representative of the general population.

### Synthesis of review outcomes

Table 2 summarises the pooled meta-analysis results for women with pre-existing mental health problems compared with women without pre-existing mental health problems for each birth outcome, showing both crude and adjusted OR/RR estimates where possible. For outcomes reported by a single study, individual study estimates are included for completeness.

**Table 2:** Summary table for crude and adjusted analyses of birth outcomes following meta-analyses

Outcome	Studies reporting OR				Studies reporting RR			
	n	cOR (95% CI), I^2^ (%)	n	aOR (95% CI), I^2^ (%)	n	cRR (95% CI), I^2^ (%)	n	aRR (95% CI), I^2^ (%)
1. Mode of birth								
**1.1 Instrumental birth** (vs normal vaginal birth)	5	0.93 (0.77 to 1.12), I^2^ 80.0	0	–	2	0.82 (0.63 to 1.08), I^2^ 55.7	3	**0.87 (0.77, 0.98), I^2^ 18.7**
**1.2 Caesarean section¶** (vs normal vaginal birth)	7	**1.53 (1.20 to 1.97), I^2^ 98.4**	1*	**1.24 (1.20 to 1.29**)	5	**1.09 (1.06 to 1.13), I^2^ 0**	5†	**1.04 (0.81 to 1.34), I^2^ 92.6**
**2. Preterm birth** (vs term birth)	18	**1.48 (1.35 to 1.62), I^2^ 83.8**	14	**1.41 (1.27 to 1.56), I^2^ 86.8**	8	**1.39 (1.25 to 1.54), I^2^ 59.9**	9†	**1.36 (1.21 to 1.51), I^2^ 66.0**
**3. Low birth weight** (vs normal birth weight)	9	**1.48 (1.31 to 1.67), I^2^ 78.7**	6	**1.28 (1.22 to 1.33), I^2^ 0**	4	**1.40 (1.13 to 1.75), I^2^ 77.6**	4	**1.32 (1.04 to 1.68), I^2^ 79.4**
**4. Small for gestational age** (vs not SGA)	10‡	**1.37 (1.13 to 1.66), I^2^ 96.7**	8‡	**1.27 (1.07 to 1.51), I^2^ 96.6**	6	**1.29 (1.15 to 1.44), I^2^ 50.3**	6†	**1.34 (1.19 to 1.51), I^2^ 54.7%**
NNU admission (vs no NNU admission)	2	**1.38 (1.05 to 1.82), I^2^ 33.0**	3	**1.44 (1.19 to 1.74), I^2^ 0**	0	–	0	–

Values in bold indicate statistical significance (p<0.05)

1.2 Caesarean section encompasses planned and unplanned caesarean section.

* Effect sizes from individual studies are reported as meta-analysis is not possible.

† As Bulik *et al* and Watson *et al*^55^ used the same dataset, only one study was included per outcome in pooled analyses to avoid double counting study participants. Watson *et al*^55^ was prioritised where effect estimates were available; Bulik *et al*^29^ was included only when the required estimates were not reported by Watson *et al.*

‡ As 44Micali *et al* and 45Momen *et al* included overlapping Danish birth cohorts, only one study was included per outcome to avoid double counting. 45Momen *et al* was prioritised due to its larger and more recent sample.

¶ Four studies Chatwin *et al*, Mantel *et al*, Vigod *et al* and, Yedid Sion *et al*^30 42 52 58^: used combined normal vaginal birth and instrumental birth as the comparison group. All other studies used only normal vaginal birth as the comparison group.

aOR, adjusted OR; aRR, adjusted risk ratio; cOR, crude OR; cRR, crude risk ratio; NNU, neonatal unit; SGA, small-for-gestational-age.

#### Mode of birth

##### Instrumental birth

Eight^32 34 39 42 48 53 55 57^ studies examined whether women with pre-existing mental health problems were more likely to have an instrumental birth than a normal vaginal birth, compared with women without pre-existing mental health problems. After pooling five crude ORs (cORs),^32 39 48 53 57^ there was no association between any pre-existing mental health problems and instrumental birth (cOR 0.93, 95% CI 0.77 to 1.12), and there was high heterogeneity between studies (I^2^=80.0%) (see online supplemental 7a). No studies reported adjusted ORs (aORs).

There was no association between any pre-existing mental health problems and instrumental birth when the two crude RRs (cRRs) were pooled (cRR 0.82, 95% CI 0.63 to 1.08, I^2^ 55.7%). However, when the adjusted RRs (aRRs) were pooled, women with any pre-existing mental health problems were less likely to have an instrumental birth than a normal vaginal birth, compared with women without pre-existing mental health problems (aRR 0.87, 95% CI 0.77 to 0.98). There was low heterogeneity between these studies (I^2^=18.7%) (see online supplemental 7b).

##### Caesarean section

**Figure 2:**
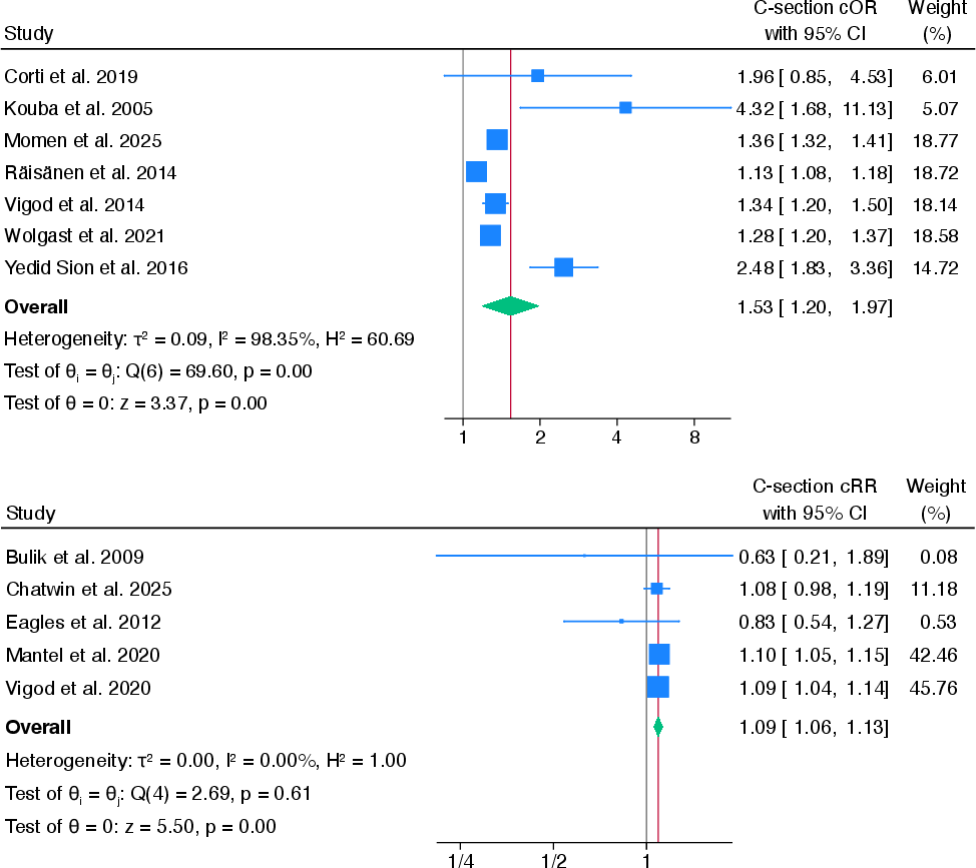
Pooled crude ORs and risk ratios for the association between any pre-existing mental health problems and caesarean section. cOR, crude OR; cRR, crude risk ratio.

13 studies^29 30 32 34 39 42 45 48 52 53 55 57 58^ examined the association between pre-existing mental health problems and caesarean compared with vaginal birth. After pooling cOR estimates from seven studies,^32 39 45 48 53 57 58^ pre-existing mental health problems were associated with caesarean section (cOR 1.53, 95% CI 1.20 to 1.97). However, there was high heterogeneity between studies (I^2^=98.4%) (see figure 2). One study^45^ reported an adjusted association between pre-existing anxiety and caesarean section (cOR 1.36, 95% CI 1.32 to 1.41; aOR 1.24, 95% CI 1.20 to 1.29) compared with women with no pre-existing mental health problems.

Results from five^29 30 34 42 52^ pooled cRRs showed that women with any pre-existing compared with no pre-existing mental health problems were more likely to have a caesarean section compared with a normal vaginal birth, although the effect size was small (cRR 1.09, 95% CI 1.06 to 1.13). There was no heterogeneity between these studies (I^2^=0%). When four^30 34 42 55^ aRRs were pooled, the association was smaller and not statistically significant (aRR 1.04, 95% CI 0.81 to 1.34), and there was high heterogeneity between studies (I^2^=92.6%) (see online supplemental 8).

3^32 39 57^ out of the 13 studies that reported on caesarean section also considered whether these were planned or unplanned. All three studies provided raw data, so only crude ORs were calculated. There was no association between pre-existing mental health problems and planned caesarean section compared with normal vaginal birth (cOR 1.82, 95% CI 0.87 to 3.81). The same three studies showed weak evidence that women with pre-existing mental health problems were also more likely to have an unplanned caesarean section than vaginal birth (cOR 1.76, 95% CI 0.94 to 3.27) as the effect was not statistically significant. There was moderate heterogeneity between studies for both planned and unplanned caesarean section, where I^2^ values were 51.8% and 54.2%, respectively (see online supplemental 9, 10).

### Preterm birth

**Figure 3:**
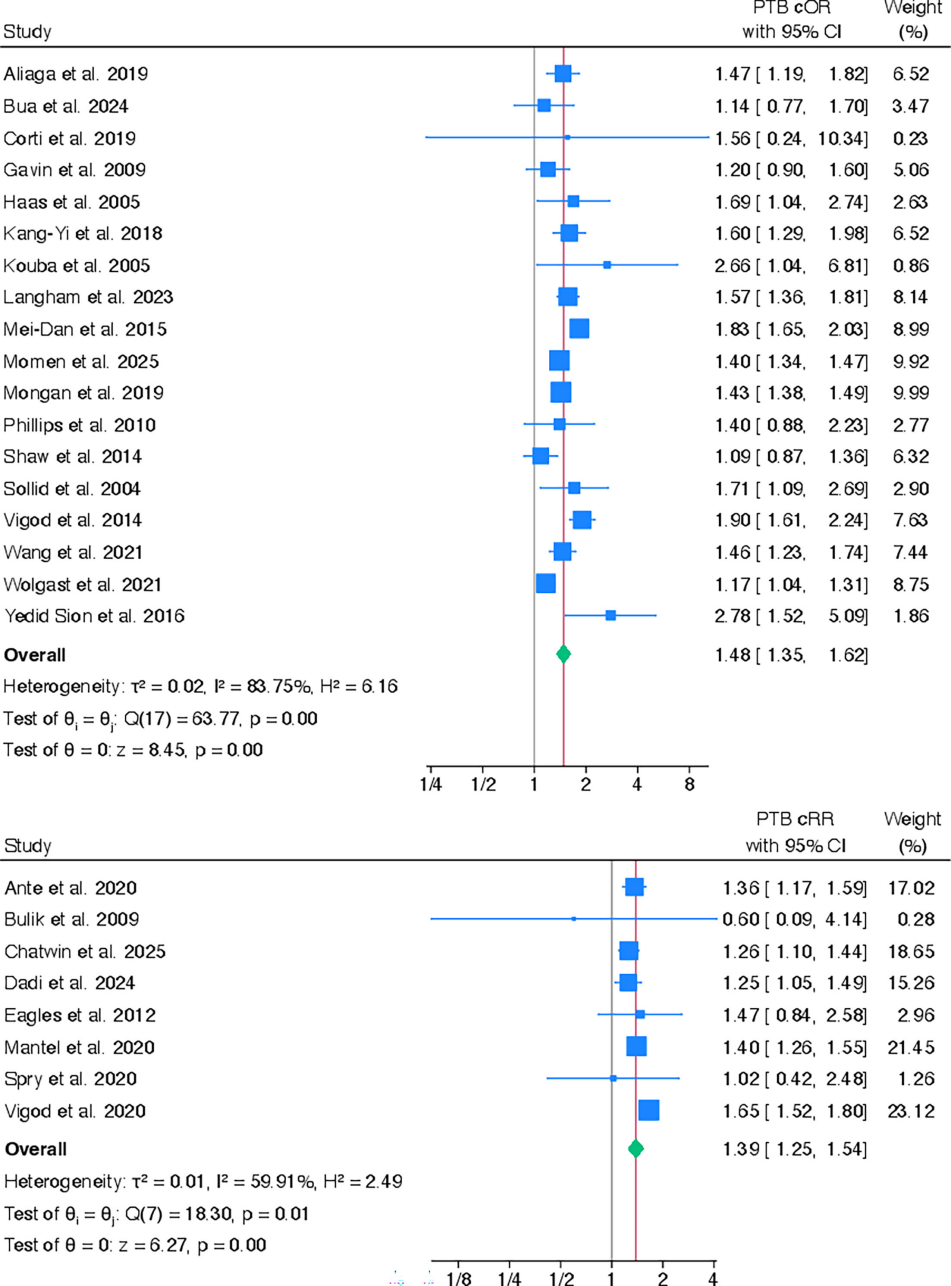
Pooled crude ORs and risk ratios for the association between any pre-existing mental health problems and PTB. cOR, crude OR; cRR, crude risk ratio; PTB, preterm birth.

27 studies^26^,^3032^ examined whether women with any pre-existing mental health problems were more likely to have PTB, compared with women without pre-existing mental health problems. The results were consistent across crude and adjusted ORs and RRs (see figure 3). Pooled results from 18 cORs showed that women with any pre-existing mental health problems were more likely to have a PTB (cOR 1.48, 95% CI 1.35 to 1.62) compared with women without any pre-existing mental health problems.^2628 32 35 36 38^,^40 43 45^ There was high heterogeneity between the studies (I^2^=83.8%). This association remained after pooling 14 adjusted estimates (aOR 1.41, 95% CI 1.27 to 1.56) and heterogeneity between studies remained high (I^2^=86.8%)^2628 36 38 40 43 45^,^47 49 50 53 54 57^ (see online supplemental 12). Results from the Egger’s test also showed no publication bias for the meta-analyses for both cORs and aORs (p=0.33 and 0.60, respectively). These results were corroborated by observing the funnel plots (see online supplemental 11, 13).

When eight cRRs^27 29 30 33 34 42 51 52^ were pooled, women with any pre-existing mental health problems were more likely to have a PTB compared with women without pre-existing mental health problems (cRR 1.39, 95% CI 1.25 to 1.54, I^2^ 59.9%). Results remained very similar after pooling the adjusted values (aRR 1.36; 95% CI 1.21 to 1.51, I^2^ 66.0%).^27 30 33 34 42 51 52 55^ Heterogeneity remained moderate for both crude and adjusted analyses.

### Low birth weight

**Figure 4:**
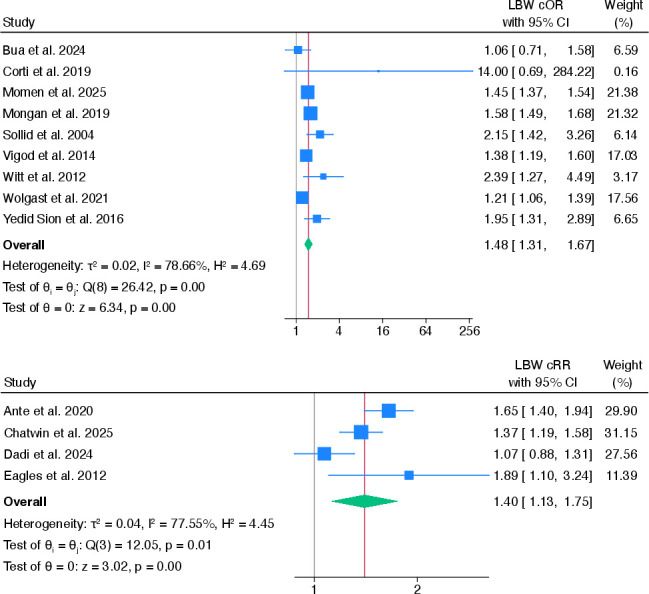
Pooled crude ORs and risk ratios for the association between any pre-existing mental health problems and LBW. cOR, crude OR; cRR, crude risk ratio; LBW, low birth weight.

13 studies examined whether women with any pre-existing mental health problems were more likely to have an infant with LBW than normal birth weight, compared with women without pre-existing mental health problems.^2728 30 32^,^34 45 46 50 53 56^ The pooled cORs showed that women with any pre-existing mental health problems were more likely to have an infant with LBW than normal birth weight, compared with women without pre-existing mental health problems (cOR 1.48, 95% CI 1.31 to 1.67).^2832 45 46 50 53 56^,^58^ The pooled aORs from six studies^28 45 46 50 56 57^ was smaller compared with the crude (aOR 1.28, 95% CI 1.22 to 1.33). There was high heterogeneity between studies for the cORs (I^2^=78.7%); however, there was no heterogeneity between studies for the adjusted ORs (I^2^=0%) (see figure 4).

Four studies^27 30 33 34^ reported both cRR and aRR estimates. Pooling these studies showed that women with any pre-existing, compared with no pre-existing, mental health problems were more likely to have an LBW infant than an infant with normal birth weight (cRR 1.40, 95% CI 1.13 to 1.75, I^2^ 77.6%; aRR 1.32, 95% CI 1.04 to 1.68, I^2^ 79.4%). Heterogeneity between studies remained high for both crude and adjusted analyses (see online supplemental 14).

### Small-for-gestational-age

**Figure 5:**
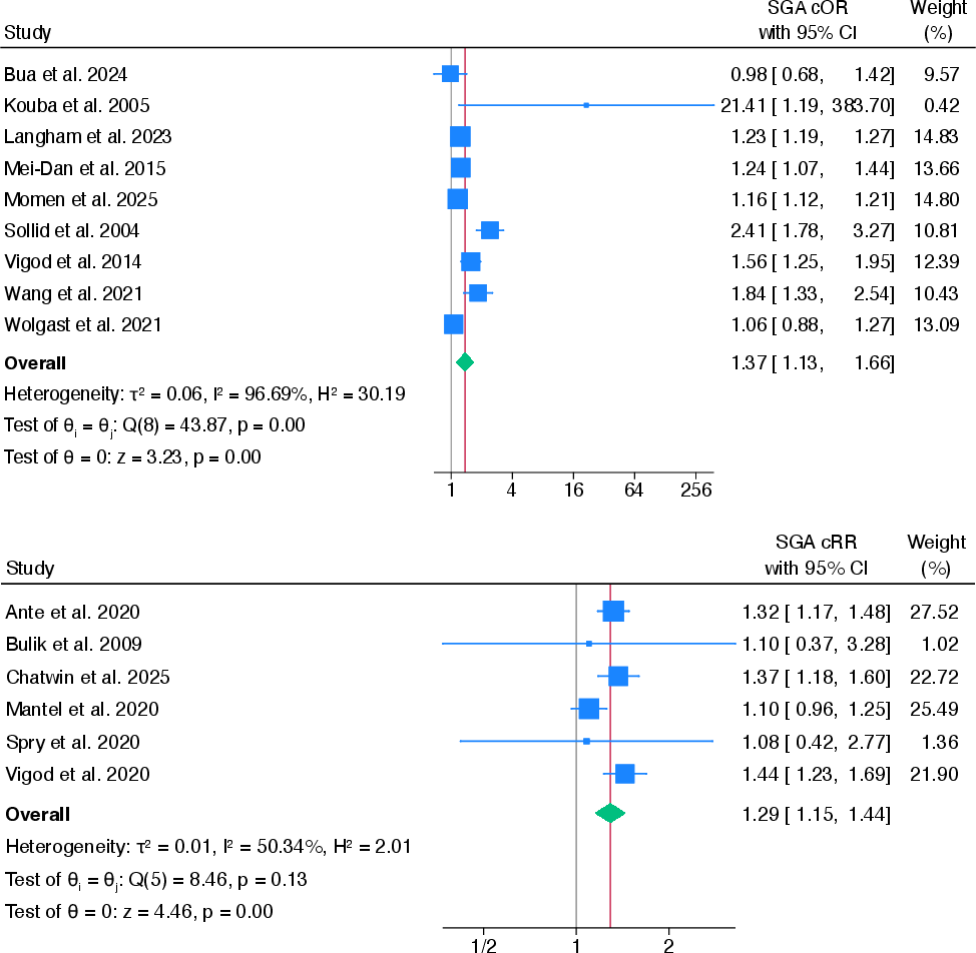
Pooled crude ORs and risk ratios for the association between any pre-existing mental health problems and having an SGA infant. cOR, crude OR; cRR, crude risk ratio; SGA, small-for-gestational-age.

18 studies examined whether women with any pre-existing, compared with no pre-existing, mental health problems were more likely to have an SGA infant.^27^,^3037 39 40 42^ One study reported HRs^37^ and another reported combined SGA and IUGR outcomes,^31^ therefore both were excluded from the meta-analysis. Pooled cOR estimates showed that women with any pre-existing mental health problems were more likely to have an SGA infant, compared with women without pre-existing mental health problems (cOR 1.37, 95% CI 1.13 to 1.66).^2839 40 43^,^45 50 53 54 57^ High heterogeneity existed between studies (I^2^=96.7%). The association between pre-existing mental health problems and having an SGA infant was smaller after pooling aORs (aOR 1.27, 95% CI 1.07 to 1.51) but heterogeneity remained high (I^2^=96.6%) (see figure 5).

After pooling six cRRs,^27 29 30 42 51 52^ results showed that women with any pre-existing, compared with no pre-existing, mental health problems were more likely to have an SGA infant than not (cRR 1.29, 95% CI 1.15 to 1.44). Additionally, this association increased slightly after pooling aRRs (aRR 1.34, 95% CI 1.19 to 1.51).^27 30 42 51 52 55^ Heterogeneity between studies remained moderate for both crude and adjusted analyses, where I^2^ values were 50.3% and 54.7%, respectively (see online supplemental 15).

### NNU admission

Three studies reported on NNU admission,^28 41 57^ where all three studies reported aORs, but only two studies reported cORs.^28 57^ Pooling the two cORs demonstrated that women with any, compared with no pre-existing mental health problems were more likely to have an infant admitted to NNU (cOR 1.38, 95% CI 1.05 to 1.82, I^2^ 33.0%). This association increased slightly after pooling aORs and heterogeneity decreased from the crude to adjusted analyses (aOR 1.44, 95% CI 1.19 to 1.74; I^2^ 0.0%) (see online supplemental 16).

### Sensitivity analyses

Sensitivity analyses were conducted to assess the robustness of the primary findings. These analyses were exploratory and should be interpreted cautiously due to the smaller number of studies contributing to each subgroup. Specifically, sensitivity analyses evaluated the consistency of pooled estimates when restricting studies by methodological quality, type of pre-existing mental health problem and characteristics of the comparison group.

#### Higher quality studies

12 out of 33 studies were categorised as higher quality as they satisfied every domain on the JBI or NOS.^27 30 34 37 40 42 45 46 50 52 53 57^ As more studies reported crude compared with adjusted estimates, all sensitivity analyses were conducted using crude estimates (see online supplemental table S4). Overall, crude associations between any pre-existing mental health problems and instrumental birth, PTB, LBW or SGA remained similar compared with the main analysis when limiting analyses to high-quality studies.

#### Type of mental health problem

##### Common mental health problems

Compared with meta-analysis results including all studies, limiting analyses to only common mental health problems led to smaller associations for pre-existing common mental health problems and PTB, LBW and SGA outcomes. However, there were no significant changes for outcomes including instrumental birth, caesarean section and NNU admission, perhaps due to limited number of studies included in the meta-analysis (see online supplemental table S5).

##### Severe mental health problems

Limiting analyses to severe mental health problems led to increased odds of PTB but decreased odds of instrumental birth and caesarean section, compared with the main analysis. However, ORs for LBW and SGA remain comparable to the main analysis. The limited number of studies contributing RRs restricted interpretation of pooled RR estimates (see online supplemental table S5).

##### Eating disorders

There were no significant changes in the crude associations between pre-existing eating disorders and either instrumental birth or PTB when limiting analysis to eating disorders. cOR associations for caesarean section, LBW and SGA became statistically insignificant after pooling only studies reporting eating disorders, perhaps due to small number of studies included. However, cRR results remained similar to the main analysis (see online supplemental table S5).

### Comparison groups

Studies with no comorbidity in the comparison group reported similar effect sizes for all outcomes compared with the main analysis. However, pooling results from articles with possible comorbidity resulted in smaller effect sizes for PTB, LBW and SGA outcomes (see online supplemental table S6).

## Discussion

### Summary of main findings

This systematic review and meta-analysis examined 33 studies exploring the association between any pre-existing mental health problems and birth outcomes. Pooled estimates were calculated for instrumental birth (n=8) and caesarean section (n=13), PTB (n=27), LBW (n=13), SGA (n=18) and NNU admission (n=3). Adjusted analyses showed that women with any pre-existing mental health problems had increased odds and risks of PTB (41% higher odds; 36% higher risk), LBW (28% higher odds; 32% higher risk), SGA (27% higher odds; 34% higher risk) and NNU admission (44% higher odds) compared with women without pre-existing mental health problems. In contrast, there was no consistent association between pre-existing mental health problems and mode of birth, including instrumental birth or caesarean section. Across outcomes, pooled estimates were accompanied by moderate to high heterogeneity, reflecting variation in study populations, definitions and timing of pre-existing mental health problems and covariate adjustment, which may reduce the precision of pooled estimates and limit their generalisability.

### Research in context

Most existing reviews examining birth outcomes in relation to maternal mental health problems focus on antenatal exposure, with limited attention to conditions that predate pregnancy. Adane *et al*^15^ examined stillbirth and infant mortality among women with pre-existing mental health problems, but no systematic review to date has assessed a broader range of obstetrical and infant outcomes in relation to pre-existing mental health problems. Montagnoli *et al*^16^ conducted a scoping review on this topic and reported tentative associations with outcomes such as caesarean section, PTB and pregnancy complications. However, there was minimal overlap with studies included in the present review, likely reflecting differences in search strategy and exposure definition. Montagnoli *et al*^16^ defined pre-existing mental health problems more broadly, using indicators such as psychiatric contact during pregnancy^59 60^ or diagnoses recorded at discharge following birth.^61^ In contrast, the present review included studies which explicitly examined mental health problems established prior to conception. This distinction was made to avoid potential misclassification and dilution of results by effects of antenatal mental health problems.

When compared with other meta-analyses of antenatal mental health problems,^3 4^ this review found similar effect sizes. However, pre-existing and antenatal mental health problems may represent epidemiologically and clinically distinct exposures, and similarities in effect size should not be interpreted as evidence of shared mechanisms. For example, Grote *et al*^4^ conducted a meta-analysis of depression during pregnancy and risks of PTB, LBW and IUGR, where they found pooled RRs of 1.39 (95% CI 1.19 to 1.61) and 1.49 (95% CI 1.25 to 1.77), respectively, for PTB and LBW. Similarly, Ghimire *et al*^3^ reported pooled RR estimates of 1.35 (95% CI 1.19 to 1.52) and 1.86 (95% CI 1.32 to 2.62) for the same two outcomes. The comparable magnitude of associations may reflect the chronic or recurrent nature of many mental health conditions, which can persist, relapse or worsen during pregnancy.^62^ These findings underscore the importance of considering mental health trajectories prior to conception, rather than focusing exclusively on antenatal exposure.

Maternal mental health problems may affect birth outcomes through a combination of biological, behavioural and social pathways, but the exact mechanisms are not yet fully understood.^63^ Dysregulation of the stress response system, particularly elevated cortisol and inflammation, can impair placental function and fetal growth, increasing the risk of PTB, LBW and SGA.^64^ Certain health disparities and lifestyle behaviours are generally more common among women with mental health problems, including smoking, lack of exercise and obesity, poor nutrition and substance use.^65^ Women with mental health problems are also more likely to have comorbid medical conditions, poor health-seeking behaviours and are less likely to access antenatal care.^66^ However, the association between mental health problems and adverse birth outcomes often persists after adjusting for these factors, thus suggesting a more complex interplay of biomedical and psychosocial factors.^67^

### Strengths and limitations

To our knowledge, this is the first systematic review and meta-analysis to comprehensively evaluate the association between pre-existing mental health problems and subsequent birth outcomes. A major strength of this review is the use of a robust, comprehensive search strategy supplemented with manual hand-searching. The search strategy was intentionally broad to maximise the likelihood of capturing all relevant studies. Another strength is the use of a clear and consistent definition for pre-existing mental health problems, which minimises the risk of misclassification and dilution of results due to antenatal mental health problems. Given the large volume of articles retrieved from databases, authors were also contacted to clarify timing of onset for mental health problems. Finally, crude and adjusted OR/RR estimates were pooled separately to observe effects of adjusting for confounders.

A limitation of this review was the exclusion of relevant studies published in languages other than English. Additionally, the broad research question led to substantial between-study heterogeneity. High heterogeneity (>75%) likely reflects differences in populations, definitions and timing of pre-existing mental health problems and mental health problems examined between studies. Sensitivity analyses restricting studies by study quality, type of pre-existing mental health problem and comparison group characteristics did not consistently reduce between-study heterogeneity. Some studies also reported either crude or adjusted effects, which introduced additional heterogeneity to the analysis and the pattern of adjustment varied between studies. Although many studies adjusted for potential confounding, there was substantial variation in the selection and reporting of covariates. As shown in online supplemental table S2, studies adjusted for a wide range of socio-demographic, obstetric, behavioural and clinical factors, with no consistent adjustment set applied across studies. Maternal age and parity were the most adjusted variables. However, other factors such as smoking, body mass index, socioeconomic status and comorbid medical conditions were occasionally included. This variability in covariate adjustment may have also contributed to residual confounding and between-study heterogeneity. Further, the majority of studies did not provide separate data on mental health problems during pregnancy or use of psychotropic medications for women with and without pre-existing mental health problems, therefore sensitivity analyses could not be conducted to evaluate the effects of these factors on overall associations.

### Implications for practice and future research

Beyond immediate health impacts, infants born to mothers with pre-existing mental health problems often face cumulative and intergenerational disadvantage. Women with mental health problems are more likely to experience stressors such as limited social support and financial hardship, which can destabilise the home environment and hinder child development.^68 69^ Poor maternal mental health may impair bonding and attachment, increasing the risk of emotional and behavioural difficulties in offspring.^70^ A review by Stein *et al*^63^ highlights long-term links between maternal mental disorders and adverse cognitive, emotional and behavioural outcomes in children. As such, addressing pre-existing mental health problems is critical to reducing these compounding risks over a child’s life. A life course approach to maternal and child health emphasises the need for early screening and intervention before pregnancy, recognising that many mental health conditions are chronic and often begin in adolescence or early adulthood.^13 71^ Therefore, interventions should aim to strengthen identification, referral and continuity of care for all women of reproductive age, with additional support for those with recurrent or a history of mental health problems.

This review identified a clear gap in the literature regarding the association between pre-existing maternal mental health problems and mode of birth and NNU admission, highlighting the need for more nuanced, population-based research. Women with pre-existing mental health conditions may be at greater risk of instrumental birth or unplanned caesarean section, potentially due to heightened anxiety or challenges engaging with maternity care. Understanding these relationships is critical to informing trauma-informed perinatal care, reducing adverse outcomes and supporting positive birth experiences. A more detailed exploration of the role of confounders and potential mediators is needed to clarify the complex pathways between pre-existing mental health and adverse birth outcomes. Specifically, antenatal mental health problems may act as important mediators on the causal pathway and should be explicitly considered in future studies. Recent evidence also suggests that risks differ between women with only pre-existing mental health conditions, those who develop symptoms during pregnancy and those with persistent or chronic symptoms spanning both periods.^39^ Clarifying these differential risks across the perinatal timeline is essential for improving early identification, tailoring interventions and enhancing care.

Given the limited number of studies on NNU admission and mode of birth, further research is needed to strengthen this evidence base. Future work should also focus on elucidating the biological, psychological and socio-behavioural mechanisms linking pre-existing mental health problems and birth outcomes, to inform the development of targeted interventions and health policies that improve outcomes for both mothers and babies.

## Conclusion

This systematic review and meta-analysis found that women with any pre-existing mental health problems had increased odds and risks of PTB, LBW and SGA compared with women without pre-existing mental health problems. Evidence for mode of birth and NNU admission was limited and inconclusive. Given that many mental health problems are chronic and predate pregnancy, greater emphasis is needed on pre-conception mental healthcare and the prevention of onset and recurrence of mental health problems. Better identification and referral pathways for mental health problems are needed for women of reproductive age, with interventions focusing on the early identification and management of women with a history of mental health problems prior to conception to support continuity of maternity care. Future research is needed to clarify associations between pre-existing maternal mental health problems and mode of birth and NNU admission, as well as elucidate the biological, psychological and socio-behavioural mechanisms underlying these associations.

## Supplementary material

10.1136/bmjopen-2025-106566 online supplemental file 1

## Data Availability

Data sharing not applicable as no datasets generated and/or analysed for this study.
