# Supplementary material for: Birth outcomes for women with pre-existing mental health problems: a systematic review and meta-analysis
Source: BMJ Open. 2026 May 29;16(5):e106566. doi: 10.1136/bmjopen-2025-106566 (PMC13223673; doi:10.1136/bmjopen-2025-106566)
Supplement: online supplemental file 1 [file bmjopen-16-5-s001.pdf]

Birth outcomes for women with pre-existing mental health problems: a systematic review and meta-analysis

Jenny Gong, Ian Henderson, Rosie Lynch, Zoe Daskalopoulou, Nia Roberts, Gracia Fellmeth, Sian Harrison, Maria Quigley, Fiona Alderdice

Supplementary Information

Table S1a: Search strategy for MEDLINE 2

1. *Table S1b: Search strategy for EMBASE* 4
2. *Table S1c: Search strategy for PsycINFO* 6
3. *Table S1d: Search strategy for CINAHL* 8
4. *Table S2: Summary of all included studies with crude and adjusted associations along with covariates adjusted for* 10

6. *Table S3: NOS quality assessment table for cohort studies and JBI Critical Appraisal Checklist for cross-sectional studies* 16

7. *Pooled crude and adjusted associations between any pre-existing mental health problems and instrumental birth* 18
   a. *Pooled crude OR/RR values for instrumental birth* 18
   b. *Pooled adjusted OR/RR values for instrumental birth* 18
8. *Pooled adjusted associations between any pre-existing mental health problems and caesarean section* 19
9. *Crude associations between any pre-existing mental health problems and planned c-section* 19
10. *Crude associations between any pre-existing mental health problems and unplanned c-section* 20
11. *Funnel plot for pooled crude associations between any pre-existing mental health problems and PTB* 20
12. *Pooled adjusted OR/RR between any pre-existing mental health problems and PTB* 21
13. *Funnel plot for pooled adjusted associations between any pre-existing mental health problems and PTB* . 22
14. *Pooled adjusted OR/RR between any pre-existing mental health problems and LBW* 22
15. *Pooled adjusted OR/RR between any pre-existing mental health problems and SGA* 23
16. *Pooled crude and adjusted ORs between any pre-existing mental health problems and NNU admission*.... 24
17. *Table S4: Summary table for sensitivity analyses of crude analyses of birth outcomes following meta-analysis (higher quality studies)* 25
18. *Table S5: Summary table for sensitivity analyses of crude analyses of birth outcomes following meta-analysis (common mental health problems, severe mental health problems, and eating disorders compared to original analysis including all studies)* 26
19. *Table S6: Summary table for sensitivity analysis of articles with no comorbidity in comparison groups and possible comorbidity, compared to the main analysis including all studies* 27
20. *Management of potential data source overlap* 28

Table S1a: Search strategy for MEDLINE

Line #	*Keywords*	*No. of Results*
1	("33386983" or "32393194" or "30927990" or "32711495" or "37842916" or "35582731" or "37576490").ui.	8
2	(depress* or anxi* or post-traumatic stress or posttraumatic stress or post-traumatic distress or posttraumatic distress or bi-polar or bipolar or dysthymi* or mania* or manic or ptsd or ptdd or phobi* or obsessive-compulsive? or ocd or bulimi* or anorexi* or bing* or personality-disorder* or suicid* or selfharm* or self-harm* or substance-misuse or substance abuse).ti,kf.	464647
3	((delusion* or paranoi* or mood or affective or neurotic or stress or reactive or combat or somatoform or somati#ation or phobi* or adjustment or dissociat* or eat* or personality or serious-mental) adj2 disorder*).ti,kf.	66476
4	((mental* or psych*) adj3 (problem* or difficult* or disorder* or ill* or health*)).ti,kf.	186228
5	(compulsive* adj1 (eat* or vomit* or purg*)).ti,kf.	77
6	exp Mental Health/	63788
7	exp Mental Disorders/	1450940
8	exp Psychiatry/	111083
9	2 or 3 or 4 or 5 or 6 or 7 or 8	1851319
10	(pregnan* or prepregnan* or preconception* or pre-conception* or antenatal or ante-natal or prenatal or pre-natal or antepartum or ante-partum or peripartum or peri-partum or trimester* or (expectant adj (mother* or mum?)) or matern* or perinatal or peri-natal or intrapart* or intra-part*).ti,kf.	508251
11	*Pregnancy/ or Pregnant Women/	46667
12	Peripartum period/	1791
13	Prenatal Care/ or Perinatal Care/ or Preconception Care/	39887
14	Maternal Health Services/	16590
15	10 or 11 or 12 or 13 or 14	536328
16	(((pregnancy or maternal or birth or childbirth or delivery or foetal or fetal or neonatal or newborn) adj3 (outcome? or complication?)) or (adverse adj2 outcome?)).ti,ab,kf.	180852
17	(((preterm or pre-term or prematur*) adj2 (birth or delivery or neonat* or newborn? or infant?)) or ("small for gestational age" or "sga low birth weight" or "low birthweight" or ptb or aga or lbw or vlbw)).ti,ab,kf.	136842
18	(caesarean or cesarean or "c section" or ((mode or instrumental or assisted) adj (birth or childbirth or delivery)) or (vaccum adj2 (birth or childbirth or delivery or extraction or obstetric*))).ti,ab,kf.	76468
19	(neonatal intensive care or newborn intensive care or nicu).ti,ab,kf.	32308
20	exp Obstetric Labor, Premature/	34687
21	Birth Weight/	44671
22	exp Infant, Low Birth Weight/	39205
23	exp Delivery, Obstetric/	92213
24	Pregnancy Outcome/	58354

25	neonatal intensive care/	6162
26	or/16-25	464351
27	9 and 15 and 26	7371
28	afghanistan/ or exp africa/ or albania/ or andorra/ or antarctic regions/ or argentina/ or exp asia, central/ or exp asia, northern/ or exp asia, southeastern/ or exp atlantic islands/ or bangladesh/ or bhutan/ or bolivia/ or borneo/ or "bosnia and herzegovina"/ or brazil/ or bulgaria/ or exp central america/ or colombia/ or "commonwealth of independent states"/ or "democratic people's republic of korea"/ or ecuador/ or exp india/ or indonesia/ or iran/ or iraq/ or jordan/ or kosovo/ or kuwait/ or lebanon/ or liechtenstein/ or macau/ or "macedonia (republic)"/ or exp melanesia/ or moldova/ or monaco/ or mongolia/ or montenegro/ or nepal/ or new guinea/ or pakistan/ or paraguay/ or peru/ or philippines/ or qatar/ or "republic of belarus"/ or exp russia/ or serbia/ or sri lanka/ or suriname/ or syria/ or taiwan/ or exp transcaucasia/ or ukraine/ or united arab emirates/ or exp ussr/ or venezuela/ or yemen/	1058424
29	organisation for economic co-operation and development/	565
30	australasia/ or exp australia/ or austria/ or exp baltic states/ or belgium/ or exp canada/ or chile/ or exp china/ or czech republic/ or europe/ or exp france/ or exp germany/ or greece/ or hungary/ or ireland/ or israel/ or exp italy/ or exp japan/ or korea/ or luxembourg/ or mexico/ or netherlands/ or new zealand/ or north america/ or poland/ or portugal/ or exp "republic of korea"/ or exp "scandinavian and nordic countries"/ or slovakia/ or slovenia/ or spain/ or switzerland/ or turkey/ or exp united kingdom/ or exp united states/	3762705
31	european union/	17812
32	developed countries/	21440
33	29 or 30 or 31 or 32	3778718
34	28 not 33	971905
35	27 not 34	6983

1. Table S1b: Search strategy for EMBASE

Line #	*Keywords*	*No. of Results*
1	("33386983" or "32393194" or "30927990" or "32711495" or "37842916" or "35582731" or "37576490").ui.	1
2	(depress* or anxi* or post-traumatic stress or posttraumatic stress or post-traumatic distress or posttraumatic distress or bi-polar or bipolar or dysthymi* or mania* or manic or ptsd or ptdd or phobi* or obsessive-compulsive? or ocd or bulimi* or anorexi* or bing* or personality-disorder* or suicid* or selfharm* or self-harm* or substance-misuse or substance abuse).ti,kf.	604764
3	((delusion* or paranoi* or mood or affective or neurotic or stress or reactive or combat or somatoform or somati#ation or phobi* or adjustment or dissociat* or eat* or personality or serious-mental) adj2 disorder*).ti,kf.	91276
4	((mental* or psych*) adj3 (problem* or difficult* or disorder* or ill* or health*)).ti,kf.	202662
5	(compulsive* adj1 (eat* or vomit* or purg*)).ti,kf.	136
6	exp *Mental Health/	68850
7	exp *Mental Disorders/	1610632
8	exp *Psychiatry/	93823
9	2 or 3 or 4 or 5 or 6 or 7 or 8	1976198
10	(pregnan* or prepregnan* or preconception* or pre-conception* or antenatal or ante-natal or prenatal or pre-natal or antepartum or ante-partum or peripartum or peri-partum or trimester* or (expectant adj (mother* or mum?)) or matern* or perinatal or peri-natal or intrapart* or intra-part*).ti,kf.	596248
11	*Pregnancy/ or Pregnant Women/	246892
12	Perinatal period/	42668
13	Prenatal Care/ or Perinatal Care/ or Prepregnancy Care/	68732
14	Maternal Health Services/	2846
15	10 or 11 or 12 or 13 or 14	706882
16	(((pregnancy or maternal or birth or childbirth or delivery or foetal or fetal or neonatal or newborn) adj3 (outcome? or complication?)) or (adverse adj2 outcome?)).ti,ab,kf.	256048
17	(((preterm or pre-term or prematur*) adj2 (birth or delivery or neonat* or newborn? or infant?)) or ("small for gestational age" or "sga low birth weight" or "low birthweight" or ptb or aga or lbw or vlbw)).ti,ab,kf.	185041
18	(caesarean or cesarean or "c section" or ((mode or instrumental or assisted) adj (birth or childbirth or delivery)) or (vaccum adj2 (birth or childbirth or delivery or extraction or obstetric*))).ti,ab,kf.	111068
19	(neonatal intensive care or newborn intensive care or nicu).ti,ab,kf.	48437
20	exp Labor/	39878
21	Birth Weight/	90132
22	exp low birth weight/	61372
23	exp obstetric delivery/	194251
24	Pregnancy Outcome/	83637
25	newborn intensive care/	27730
26	or/16-25	680952
27	9 and 15 and 26	9672

28	afghanistan/ or exp africa/ or albania/ or andorra/ or antarctic regions/ or argentina/ or exp asia, central/ or exp asia, northern/ or exp asia, southeastern/ or exp atlantic islands/ or bangladesh/ or bhutan/ or bolivia/ or borneo/ or "bosnia and herzegovina"/ or brazil/ or bulgaria/ or exp central america/ or colombia/ or "commonwealth of independent states"/ or "democratic people's republic of korea"/ or ecuador/ or exp india/ or indonesia/ or iran/ or iraq/ or jordan/ or kosovo/ or kuwait/ or lebanon/ or liechtenstein/ or macau/ or "macedonia (republic)"/ or exp melanesia/ or moldova/ or monaco/ or mongolia/ or montenegro/ or nepal/ or new guinea/ or pakistan/ or paraguay/ or peru/ or philippines/ or qatar/ or "republic of belarus"/ or exp russia/ or serbia/ or sri lanka/ or suriname/ or syria/ or taiwan/ or exp transcaucasia/ or ukraine/ or united arab emirates/ or exp ussr/ or venezuela/ or yemen/	1430716
29	organisation for economic co-operation and development/	2774
30	australasia/ or exp australia/ or austria/ or exp baltic states/ or belgium/ or exp canada/ or chile/ or exp china/ or czech republic/ or europe/ or exp france/ or exp germany/ or greece/ or hungary/ or ireland/ or israel/ or exp italy/ or exp japan/ or korea/ or luxembourg/ or mexico/ or netherlands/ or new zealand/ or north america/ or poland/ or portugal/ or exp "republic of korea"/ or exp "scandinavian and nordic countries"/ or slovakia/ or slovenia/ or spain/ or switzerland/ or turkey/ or exp united kingdom/ or exp united states/	4038717
31	european union/	31487
32	developed country/	35727
33	29 or 30 or 31 or 32	4072163
34	28 not 33	1264627
35	27 not 34	9110
36	conference*.pt. or conference abstract/	5739095
37	35 not 36	7456

2. Table S1c: Search strategy for PsycINFO

Line #	*Keywords*	*No. of Results*
1	("33386983" or "32393194" or "30927990" or "32711495" or "37842916" or "35582731" or "37576490").ui.	0
2	(depress* or anxi* or "post-traumatic stress" or "posttraumatic stress" or "post-traumatic distress" or "posttraumatic distress" or bi-polar or bipolar or dysthymi* or mania* or manic or ptsd or ptdd or phobi* or obsessive-compulsive? or ocd or bulimi* or anorexi* or bing* or personality-disorder* or suicid* or selfharm* or self-harm* or substance-misuse or "substance abuse").ti.	329759
3	((delusion* or paranoi* or mood or affective or neurotic or stress or reactive or combat or somatoform or somati#ation or phobi* or adjustment or dissociat* or eat* or personality or serious-mental) adj2 disorder*).ti.	56793
4	((mental* or psych*) adj3 (problem* or difficult* or disorder* or ill* or health*)).ti.	126892
5	(compulsive* adj1 (eat* or vomit* or purg*)).ti.	86
6	exp Mental Health/	93633
7	exp Mental Disorders/	1087050
8	exp Psychiatry/	57631
9	2 or 3 or 4 or 5 or 6 or 7 or 8	1278530
10	(pregnan* or prepregnan* or preconception* or pre-conception* or antenatal or ante-natal or prenatal or pre-natal or antepartum or ante-partum or peripartum or peri-partum or trimester* or (expectant adj (mother* or mum?)) or matern* or perinatal or peri-natal or intrapart* or intra-part*).ti.	52692
11	*Pregnancy/ or Expectant Mothers/	22734
12	Perinatal Period/	4342
13	Prenatal Care/ or Perinatal Care/ or Preconception Care/	2305
14	10 or 11 or 12 or 13	59992
15	(((pregnancy or maternal or birth or childbirth or delivery or foetal or fetal or neonatal or newborn) adj3 (outcome? or complication?)) or (adverse adj2 outcome?)).ti,ab,id.	16122
16	(((preterm or pre-term or prematur*) adj2 (birth or delivery or neonat* or newborn? or infant?)) or ("small for gestational age" or "sga low birth weight" or "low birthweight" or ptb or aga or lbw or vlbw)).ti,ab,id.	10854
17	(caesarean or cesarean or "c section" or ((mode or instrumental or assisted) adj (birth or childbirth or delivery)) or (vaccum adj2 (birth or childbirth or delivery or extraction or obstetric*))).ti,ab,id.	2459
18	("neonatal intensive care" or "newborn intensive care" or nicu).ti,ab,id.	3116
19	exp Obstetrics/	3664
20	exp Caesarean birth/ or exp Premature Birth/	7079
21	exp Birth Weight/	3594
22	pregnancy outcomes/	1545
23	exp Neonatal Intensive Care/	2036

24	15 or 16 or 17 or 18 or 19 or 20 or 21 or 22 or 23	33527
25	9 and 14 and 24	2885
26	afghanistan/ or exp africa/ or albania/ or andorra/ or "antarctic regions"/ or argentina/ or exp "asia, central"/ or exp "asia, northern"/ or exp "asia, southeastern"/ or exp "atlantic islands"/ or bangladesh/ or bhutan/ or bolivia/ or borneo/ or "bosnia and herzegovina"/ or brazil/ or bulgaria/ or exp "central america"/ or colombia/ or "commonwealth of independent states"/ or "democratic people's republic of korea"/ or ecuador/ or exp india/ or indonesia/ or iran/ or iraq/ or jordan/ or kosovo/ or kuwait/ or lebanon/ or liechtenstein/ or macau/ or "macedonia (republic)"/ or exp melanesia/ or moldova/ or monaco/ or mongolia/ or montenegro/ or nepal/ or "new guinea"/ or pakistan/ or paraguay/ or peru/ or philippines/ or qatar/ or "republic of belarus"/ or exp russia/ or serbia/ or "sri lanka"/ or suriname/ or syria/ or taiwan/ or exp transcaucasia/ or ukraine/ or "united arab emirates"/ or exp ussr/ or venezuela/ or yemen/	7547
27	exp australia/ or austria/ or exp "baltic states"/ or belgium/ or exp canada/ or chile/ or exp china/ or "czech republic"/ or europe/ or exp france/ or exp germany/ or greece/ or hungary/ or ireland/ or israel/ or exp italy/ or exp japan/ or korea/ or luxembourg/ or mexico/ or netherlands/ or "new zealand"/ or "north america"/ or poland/ or portugal/ or exp "republic of korea"/ or exp "scandinavian and nordic countries"/ or slovakia/ or slovenia/ or spain/ or switzerland/ or turkey/ or exp "united kingdom"/ or exp "united states"/	2153
28	exp Developed Countries/	1544
29	27 or 28	3697
30	26 not 29	7546
31	25 not 30	2883

3. Table S1d: Search strategy for CINAHL

Line #	*Keywords*
1	((((TI depress*) OR (TI anxi*) OR (TI "post N1 traumatic stress") OR (TI "posttraumatic stress") OR (TI "post N1 traumatic distress") OR (TI "posttraumatic distress") OR (TI bi N1 polar) OR (TI bipolar) OR (TI dysthymi*) OR (TI mania*) OR (TI manic) OR (TI ptsd) OR (TI ptdd) OR (TI phobi*) OR (TI obsessive N1 compulsive*) OR (TI ocd)OR (TI bulimi*) OR (TI anorexi*) OR (TI bing*) OR (TI personality N1 disorder*) OR (TI suicid*) OR (TI selfharm*) OR (TI self N1 harm*) OR (TI substance N1 misuse) OR (TI "substance abuse")) OR (((TI delusion*) OR (TI paranoi*) OR (TI mood) OR (TI affective) OR (TI neurotic) OR (TI stress) OR (TI reactive) OR (TI combat) OR (TI somatoform) OR (TI somatifation) OR (TI phobi*) OR (TI adjustment) OR (TI dissociat*) OR (TI eat*) OR (TI personality) OR (TI serious N1 mental)) N2 (TI disorder*)) OR (((TI mental*) OR (TI psych*)) N3 ((TI problem*) OR (TI difficult*) OR (TI disorder*) OR (TI ill*) OR (TI health*))) OR ((TI compulsive*) N1 ((TI eat*) OR (TI vomit*) OR (TI purg*))) OR (MH "Mental Health") OR (MM "Mental Disorders+") OR (MM Psychiatry))
2	AND
3	(((TI pregnan*) OR (TI prepregnan*) OR (TI preconception*) OR (TI pre N1 conception*) OR (TI antenatal) OR (TI ante N1 natal) OR (TI prenatal) OR (TI pre N1 natal) OR (TI antepartum) OR (TI ante N1 partum) OR (TI peripartum) OR (TI peri N1 partum) OR (TI trimester*) OR ((TI expectant) W1 ((TI mother*) OR (TI mum*))) OR (TI matern*)OR (TI perinatal) OR (TI peri N1 natal) OR (TI intrapart*) OR (TI intra N1 part*)) OR (MH "Pregnancy") OR (MH "Expectant Mothers") OR (MH "Perinatal period") OR (MH "Perinatal Care") OR (MH "Prepregnancy care") OR (MH "Maternal Health Services"))
4	AND
5	(((((TI pregnancy OR AB pregnancy) OR (TI maternal OR AB maternal) OR (TI birth OR AB birth) OR (TI childbirth OR AB childbirth) OR (TI delivery OR AB delivery) OR (TI foetal OR AB foetal) OR (TI fetal OR AB fetal OR SU fetal) OR (TI neonatal OR AB neonatal) OR (TI newborn OR AB newborn)) N3 ((TI outcome* OR AB outcome*) OR (TI complication* OR AB complication*))) OR ((TI adverse OR AB adverse) N2 (TI outcome* OR AB outcome*))) OR ((((TI preterm OR AB preterm) OR (TI pre N1 term OR AB pre N1 term) OR (TI prematur* OR AB prematur*)) N2 ((TI birth OR AB birth) OR (TI delivery OR AB delivery) OR (TI neonat* OR AB neonat*) OR (TI newborn* OR AB newborn*) OR (TI infant* OR AB infant*))) OR ((TI "small for gestational age" OR AB "small for gestational age") OR (TI "low birth weight" OR AB "low birth weight") OR (TI "low birthweight" OR AB "low birthweight") OR (TI ptb OR AB ptb) OR (TI sga OR AB sga) OR (TI lbw OR AB lbw) OR (TI vlbw OR AB vlbw)) OR ((TI caesarean OR AB caesarean) OR (TI cesarean OR AB cesarean) OR (TI "c section" OR AB "c section") OR (((TI mode OR AB mode) OR (TI instrumental OR AB instrumental) OR (TI assisted OR AB assisted)) W1 ((TI birth OR AB birth) OR (TI childbirth OR AB childbirth) OR (TI delivery OR AB delivery))) OR ((TI vaccum OR AB vaccum) N2 ((TI birth OR AB birth) OR (TI childbirth OR AB childbirth) OR (TI delivery OR AB delivery) OR (TI extraction OR AB extraction) OR (TI obstetric* OR AB obstetric*)))) OR ((TI "neonatal intensive care" OR AB "neonatal intensive care") OR (TI "newborn intensive care" OR AB "newborn born intensive care") OR (TI nicu OR AB nicu)) OR (MH "Delivery Obstetric") OR (MH "Obstetric Labor Premature") OR (MH "Birth Weight") OR (MH "Infant Low Birth Weight") OR (MH "Delivery Obstetric") OR (MH "Pregnancy Outcomes") OR (MH "Intensive Care, Neonatal") OR (MH "Intensive Care Units, Neonatal")))
6	NOT
7	(((MH afghanistan) OR (MH "Africa+") OR (MH albania) OR (MH andorra) OR (MH "antarctic regions+") OR (MH argentina) OR (MH "asia central+") OR (MH "asia northern+") OR (MH "asia southeastern+") OR (MH "atlantic islands+") OR (MH bangladesh) OR (MH bhutan) OR (MH bolivia) OR (MH borneo) OR (MH "bosnia and herzegovina") OR (MH brazil) OR (MH bulgaria) OR (MH "central America+") OR (MH colombia) OR (MH "commonwealth of independent states+") OR (MH "democratic people republic of korea") OR (MH ecuador) OR (MH india) OR (MH indonesia) OR (MH iran) OR (MH iraq) OR (MH jordan) OR (MH kosovo) OR (MH kuwait) OR (MH lebanon) OR (MH liechtenstein) OR (MH macau) OR (MH "macedonia (republic)") OR (MH melanesia) OR (MH moldova)

	OR (MH monaco)OR (MH mongolia) OR (MH montenegro) OR (MH nepal) OR (MH " guinea") OR (MH pakistan) OR (MH paraguay) OR (MH peru) OR (MH philippines) OR (MH qatar) OR (MH "republic of belarus") OR (MH russia) OR (MH serbia) OR (MH "sri lanka") OR (MH suriname) OR (MH syria) OR (MH taiwan) OR (MH transcaucasia) OR (MH ukraine) OR (MH "united arab emirates") OR (MH ussr) OR (MH venezuela) OR (MH yemen)) NOT ((MH "Organisation for Economic Co-Operation and Development") OR (MH australia) OR (MH austria) OR (MH "baltic states+") OR (MH belgium) OR (MH canada) OR (MH chile) OR (MH china) OR (MH "czech republic") OR (MH europe) OR (MH france) OR (MH germany) OR (MH greece) OR (MH hungary) OR (MH ireland) OR (MH israel) OR (MH italy) OR (MH japan) OR (MH korea)OR (MH luxembourg) OR (MH mexico) OR (MH netherlands) OR (MH " new zealand") OR (MH "north America+") OR (MH poland) OR (MH portugal) OR (MH "republic of korea") OR (MH "scandinavian and nordic countries+") OR (MH slovakia) OR (MH slovenia) OR (MH spain) OR (MH switzerland) OR (MH turkey) OR (MH "united kingdom+") OR (MH "united states") OR (MH "european union") OR (MH "Developed Countries")))

4. Table S2: Summary of all included studies with crude and adjusted associations along with covariates adjusted for

Author	Mental health problem(s) examined	Birth outcomes examined	Crude RR/OR	Adjusted RR/OR	Covariates adjusted for
1. Aliaga et al. 2019	Affective disorders (n=1,631 women)	PTB	*cOR 1.47 (1.19-1.82)*	aOR 1.32 (1.04-1.64)	Divorce, smoking, psychoactive substance use, abuse of alcohol and tobacco, history of antidepressant intake and use of antipsychotics during pregnancy
2. Ante et al. 2020	AN (n=1,842 women)	PTB	cRR 1.36 (1.17–1.59)	aRR 1.35 (1.15–1.58)	Maternal age, parity, comorbid mental disorders, preexisting metabolic disorders, substance use, socioeconomic status, place of residence, and time period at delivery
		LBW	cRR 1.65 (1.40–1.94)	aRR 1.67 (1.41–1.97)	
		SGA	cRR 1.32 (1.18–1.49)	aRR 1.47 (1.30–1.67)	
3. Bua et al. 2024	Composite depression or anxiety (n=525 women)	PTB	*cOR 1.14 (0.75-1.66)*	aOR 1.09 (0.70-1.70)	Maternal age, educational level, parity, child sex, country of birth, region of delivery, and the predicted equivalized household income
		LBW	*cOR 1.06 (0.70-1.55)*	aOR 1.00 (0.66-1.52)	
		SGA	*cOR 0.98 (0.67-1.40)*	aOR 0.97 (0.66-1.42)	
		NNU admission	*cOR 1.14 (0.75-1.68)*	aOR 1.09 (0.72-1.65)	
4. Bulik et al. 2009	AN (n=35 women)	C-section	cRR 0.63 (0.21-1.9)	aRR 1.0 (0.36-2.8)	Gestational age, maternal age, income, education, parity, smoking, pre-pregnancy BMI and gestational weight gain
		PTB	cRR 0.60 (0.088-4.2)	aRR 0.83 (0.12-5.9)	
		SGA	cRR 1.1 (0.36-3.2)	aRR 0.87 (0.3-2.5)	
5. Chatwin et al. 2025	AN (n=4,957 women)	C-section	cRR 1.08 (0.98-1.18)	aRR 0.84 (0.76-0.93)	Maternal age at delivery, primiparity, maternal marital status, maternal highest education level, calendar year of delivery, prior comorbid mental disorders (yes/no), and number of non-psychiatric hospital visits in the two years pre-conception (0, 1, 2, 3, ≥4).
		PTB	cRR 1.26 (1.10-1.44)	aRR 1.17 (1.00-1.36)	
		LBW	cRR 1.37 (1.19-1.58)	aRR 1.22 (1.04-1.43)	
		SGA	cRR 1.37 (1.18-1.60)	aRR 1.37 (1.16-1.62)	
6. Ciesielski et al. 2015	History of depression/anxiety/OCD[6] with/ without diagnosis during pregnancy (n=74 women)	Poor fetal growth (SGA or IUGR or both)	History of psychiatric diagnosis but no active diagnosis during pregnancy cOR 0.93 (0.32-2.67), History of psychiatric diagnosis and active diagnosis during pregnancy cOR 3.73 (2.09-6.68)	History of psychiatric diagnosis but no active diagnosis during pregnancy aOR 0.45 (0.09-2.35), History of Psychiatric diagnosis and active diagnosis during pregnancy aOR 2.87 (1.15-7.20)	Antidepressant medication, tobacco use, alcohol use, recreational drug use, first pregnancy vs. not first pregnancy, race (white, black, asian, or other race/unknown), insurance (public, private, military, self-pay), maternal age (<20, 20–34, and >34 years), maternal height, and maternal weight

7. Corti et al. 2019	History of depression/anxiety (n=42 women)	Mode of birth (instrumental, planned and unplanned c-section, overall c-section)	*Instrumental birth: cOR 2.75 (0.18-39.71), planned c-section: cOR 1.77 (0.58-5.20), unplanned c-section: cOR 2.16 (0.75-6.09), overall c-section: cOR 1.96 (0.85-4.55)*	N/A	N/A
		PTB	*cOR 1.56 (0.22-9.66)*	N/A	N/A
		LBW	*cOR 14.00 (0.69, 284.22)*	N/A	N/A
8. Dadi et al. 2024	One or more of any mental health disorders	PTB	cRR 1.25 (1.05-1.49)	aRR 1.20 (1.01-1.41)	Maternal age, parity, pre-existing diabetes, pre-existing hypertension, gestational diabetes, pre-eclampsia or eclampsia; ANC visits, IUGR, smoking, alcohol consumption during pregnancy, administrative health districts, and history of adversity-related admission
		LBW	cRR 1.07 (0.87-1.30)	aRR 1.03 (0.84-1.25)	
9. Eagles et al. 2012	AN (n=134 women, 230 births)	Instrumental birth	cRR 0.66 (0.44-0.98)	aRR 0.67 (0.44-1.01)	BMI, smoking, social class, marital status, pre-eclampsia, antepartum haemorrhage, induction of labour
		C-section	cRR 0.83 (0.54–1.27)	aRR 0.86 (0.54-1.36)	
		PTB	cRR 1.47 (0.84-2.59)	aRR 1.30 (0.71-2.39)	
		LBW	cRR 1.89 (1.10-3.23)	aRR 1.61 (0.89-2.90)	
10. Gavin et al. 2009	Depression (n=624 women)	PTB	cOR 1.2 (0.9–1.6)	N/A	N/A
11. Haas et al. 2005	Depression (n=184 women)	PTB	cOR 1.69 (1.04-2.74)	aOR 1.09 (0.60-1.96)	Adjusted for age, country of birth, race/ethnicity, level of education, parity, site of care, body mass index, physical function prior to pregnancy, chronic health conditions prior to pregnancy, level of exercise during the month prior to pregnancy, smoking status prior to pregnancy, smoking status during pregnancy, physical function during pregnancy, depressive symptoms during pregnancy, eclampsia or preeclampsia during pregnancy, gestational diabetes, other pregnancy complications, use of illicit drugs during pregnancy, and inadequate prenatal care
12. Jensen et al. 2013	Depression (3,287 women had diagnosis of depression before pregnancy)	SGA	Unspecified	Depression diagnosis before pregnancy cHR 0.99 (0.90–1.10)	HRs are adjusted for maternal age, smoking status, social status, calendar year, sex of newborn, and use of antiepileptics, antipsychotics, and other types of medication

13. Kang-Yi et al. 2018	4,965 women with any psychiatric treatment one year prior to pregnancy	PTB	*Psychiatric treatment 1 yr pre-preg: cOR 1.60 (1.29-1.98);* Depression one year prior to pregnancy: cOR 1.14 (0.87-1.50)	Psychiatric treatment: aOR 1.61 (1.30-1.97), Depression: aOR 1.09 (0.82-1.44)	For psychiatric treatment one year prior: age at delivery, race/ethnicity, and chronic illness status one year prior to and during pregnancy. For depression: additionally adjusted for substance abuse, smoking, anticonvulsant use, and benzodiasepine use
14. Kouba et al. 2005	AN, BN, EDNOS (n=49)	Instrumental birth, Planned C-section, Unplanned C-section, C-section overall	*Instrumental birth cOR 0.66 (0.14-2.55); Planned c-section cOR 7.19 (1.56-44.01); Unplanned c-section cOR 3.45 (1.20-10.13); C-section overall: cOR 4.32 (1.69-11.21)*	N/A	N/A
		PTB	*cOR 2.66 (1.04-6.81)*	N/A	N/A
		SGA	*cOR 21.41 (1.19, 383.70)*	N/A	N/A
15. Langham et al. 2023	General pre-existing mental illness requiring secondary mental health contact (n= 151,770)	PTB	cOR 1.57 (1.36–1.81)	aOR 1.53 (1.35–1.73)	Maternal age, parity and previous caesarean section, maternal ethnicity, socioeconomic deprivation, pre-existing diabetes, pre-existing hypertension, gestational diabetes, and pre-eclampsia or eclampsia.
		SGA	cOR 1.23 (1.19–1.27)	aOR 1.34 (1.30–1.37)	
16. Latendresse et al. 2015	Depression (6.5% of total sample - weighted)	NNU admission	Not reported	Before pregnancy aOR 1.66 (1.12-2.45)	Ethnicity, age, race, BMI, alcohol and tobacco use during pregnancy, IPV, stressful life events, poverty, education, marital status, history of previous PTB
17. Mantel et al. 2020*	EDNOS (n=6,987), AN (n=4938), BN (n=2629)	Instrumental birth, C-section overall	Instrumental birth: AN cRR 0.9 (0.8-1.0), BN cRR 0.9 (0.8-1.1), EDNOS cRR 0.9 (0.8-0.9).<br>C-section overall: AN cRR 1.0 (0.9-1.1), BN cRR 1.1 (1.0-1.2), EDNOS cRR 1.1 (1.0-1.1)	Instrumental birth: AN aRR 0.8 (0.7-0.9), BN aRR 0.9 (0.8-1.0), EDNOS aRR 0.9 (0.8-0.9)<br>C-section overall: AN aRR 1.0 (0.9-1.1), BN aRR 1.0 (1.0-1.1), EDNOS RR 1.1 (1.1-1.2)	Smoking status, parity, age and year of birth
		PTB	AN cRR 1.6 (1.5-1.8), BN cRR 1.2 (1.1-1.5), EDNOS cRR 1.4 (1.3-1.6)	AN aRR 1.6 (1.4-1.8), BN aRR 1.2 (1.0-1.5), ENDOS aRR 1.4 (1.3-1.6)	Smoking status, parity, and age
		SGA	AN cRR 1.3 (1.1-1.6), BN cRR 1.1 (0.9-1.4), EDNOS cRR 1.1 (1.0-1.3)	AN: aRR 1.3 (1.1-1.6), BN: aRR 1.1 (0.9-1.4), EDNOS: aRR 1.1 (0.9-1.2)	Smoking status, parity, and age
18. Mei-Dan et al. 2015	Major Depressive Disorder (n=3,724 unique women with 4,487 deliveries) Bipolar	PTB	MDD cOR 1.83 (1.65-2.03); BD cOR 1.93 (1.67-2.23)	MDD aOR 1.91 (1.72-2.13); BD aOR 1.95 (1.68-2.26)	Maternal age, parity, infant sex, prepregnancy obesity, substance/alcohol use disorder, diabetes mellitus, hypertension,

Abbreviations:
EDNOS = Eating disorder not otherwise specified

	Disorder (n=1859 women, 2124 deliveries))	SGA	MDD cOR 1.24 (1.07-1.44); BD cOR 1.18 (0.95-1.47)	MDD aOR 1.22 (1.05-1.42); BD aOR 1.15 (0.92-1.43)	venous thromboembolism, gestational diabetes mellitus, gestational hypertension, preeclampsia/eclampsia
19. Micali et al. 2015	AN (n=1,262)	SGA	*Past onset AN cOR 1.45 (1.23, 1.71)*	Past onset AN aOR 1.43 (1.21-1.68)	Gestational age and gender, maternal age, parity, maternal social status, alcohol use in pregnancy
20. Momen et al. 2025	Any mental health disorder (n= 48,646), Schizophrenia (n=3,332), Mood disorders (n=13,586), Anxiety related disorders (n=29,974), Eating disorders (n=8,713), Personality disorders (n=12,175)	C-section	Schizophrenia related disorders cOR 1.35 (1.23-1.48), Mood disorders cOR 1.38 (1.32-1.45), Anxiety-related disorders cOR 1.36 (1.32-1.41), Eating disorders cOR 1.04 (0.98-1.11), Personality disorders cOR 1.33 (1.27-1.40)	Schizophrenia related disorders aOR 1.13 (1.00-1.29), Mood disorders aOR 1.18 (1.11-1.24), Anxiety-related disorders aOR 1.24 (1.20-1.29), Eating disorders aOR 0.91 (0.85-0.98), Personality disorders aOR 1.18 (1.10-1.26)	Adjusted for maternal age at delivery; parity; marital status; highest education; calendar year of delivery; prior comorbid mental disorders.
		PTB	Schizophrenia related disorders cOR 1.54 (1.34-1.78), Mood disorders cOR 1.46 (1.36-1.57), Anxiety-related disorders cOR 1.40 (1.34-1.47), Eating disorders cOR 1.26 (1.14-1.38), Personality disorders cOR 1.43 (1.32-1.54)	Schizophrenia related disorders aOR 1.13 (0.93-1.38), Mood disorders aOR 1.34 (1.23-1.46), Anxiety-related disorders aOR 1.31 (1.24-1.38), Eating disorders aOR 1.19 (1.08-1.32), Personality disorders aOR 1.24 (1.12-1.37)	
		LBW	Schizophrenia related disorders cOR 1.61 (1.37-1.88), Mood disorders cOR 1.49 (1.37-1.62), Anxiety-related disorders cOR 1.45 (1.37-1.54), Eating disorders cOR 1.25 (1.12-1.40), Personality disorders cOR 1.51 (1.38-1.65)	Schizophrenia related disorders cOR 1.10 (0.88-1.38), Mood disorders cOR 1.32 (1.20-1.46), Anxiety-related disorders aOR 1.28 (1.20-1.36) Eating disorders cOR 1.17 (1.03-1.32), Personality disorders cOR 1.23 (1.10-1.38)	
		SGA	Schizophrenia related disorders cOR 1.27 (1.14-1.42), Mood disorders cOR 1.12 (1.06-1.19), Anxiety related disorders cOR 1.16 (1.11-1.20), Eating disorders cOR 1.18 (1.10-1.27), Personality disorders cOR 1.22 (1.15-1.30)	Schizophrenia related disorders cOR 1.07 (0.93-1.24), Mood disorders cOR 1.02 (0.95-1.09), Anxiety related disorders aOR 1.03 (0.99-1.07), Eating disorders cOR 1.13 (1.05-1.22), Personality disorders cOR 1.10 (1.02-1.19)	
21. Mongan et al. 2019	Any of the following mental health disorders: schizophrenia, bipolar	PTB	cOR 1.43 (1.37-1.48)	aOR 1.31 (1.25-1.37)	Antenatal smoking, antenatal alcohol status, maternal age, BMI, IPV, employment status,

	disorder, OCD, eating disorders, depression, psychosis (n= 26,547 singleton pregnancies)	LBW	cOR 1.58 (1.49-1.68)	aOR 1.29 (1.21-1.38)	parenting status (lone parent/not lone parent), fetal abnormality registered during pregnancy (present/not present), hypertensive disorder (pre-eclampsia, chronic hypertension or gestational hypertension) registered during pregnancy (present/not present)
22. Phillips et al. 2010	Depression - above or equal 23 on CES-D (n=290 births, 274 mothers)	PTB	*cOR 1.40 (0.86-2.19)*	aOR 1.40 (0.88–2.24)	Age at delivery, marital status, education, body mass index, cigarette smoking, parity, mother born preterm, and any comorbid condition (diabetes, hypertension, or thyroid conditions)
23. Räisänen et al. 2014	Depression (n= 16,712 women)	Instrumental birth (forceps and vacuum), C-section	*Instrumental cOR 1.06 (1.00-1.13), C-section cOR 1.13 (1.08-1.18)*	N/A	N/A
24. Shaw et al. 2014	PTSD (n=1,128 deliveries)	PTB	cOR 1.09 (0.87-1.36)	aOR 1.06 (0.84-1.34)	Age, race, multiple gestation, and deployment history
25. Sollid et al. 2004	AN, BN, and EDNOS (n= 302 women, 504 births)	PTB	*cOR 1.71 (1.09-2.69)*	aOR 1.7 (1.1-2.6)	Maternal age, parity, marital status, sex of child
		LBW	*cOR 2.15 (1.41-3.25)*	aOR 2.2 (1.4-3.2)	
		SGA	*cOR 2.41 (1.77-3.25)*	aOR 1.8 (1.3-2.4)	
26. Spry et al. 2020	Common mental health disorders (anxiety and depression) (n=126 adolescents, 82 young adults, 162 total)	PTB	cRR 1.02 (0.42-2.49)	aRR 1.04 (0.40-2.70)	Parents completing high school, ethnicity, overweight, underweight, binge drinking and tobacco smoking in adolescence
		SGA	cRR 1.08 (0.42-2.76)	aRR 0.96 (0.38-2.40)	
27. Vigod et al. 2020	Schizophrenia (n= 4,279 deliveries)	C-section overall	*cRR 1.09 (1.04-1.14)*	N/A	N/A
		PTB	cRR 1.65 (1.51-1.79)	aRR 1.64 (1.51-1.79)	Maternal age at delivery, parity, neighbourhood income quintile, region of residence, infant sex, and year of childbirth
		SGA	cRR 1.44 (1.23-1.69)	aRR 1.40 (1.20-1.64)	
28. Vigod et al. 2014	Schizophrenia (n= 1,628 deliveries to 1,391 women)	Instrumental birth	*cOR 0.75 (0.62-0.90)*	N/A	N/A
		C-section	*cOR 1.34 (1.20-1.50)*	N/A	N/A
		PTB	cOR 1.90 (1.61–2.24)	aOR 1.75 (1.46–2.08)	Maternal age (continuous in years), parity (0, 1, 2+), income quintile, community size, pre-pregnancy diabetes mellitus, pre-pregnancy hypertension, pre-pregnancy thromboembolic disease and infant sex
		SGA	cOR 1.56 (1.25–1.95)	aOR 1.49 (1.19–1.86)	
		LBW	*cOR 1.38 (1.19-1.59)*	N/A	N/A
29. Wang et al. 2021	Those who used antipsychotics before pregnancy	PTB	cOR 1.46 (1.23- 1.74)	aOR 1.47 (1.23-1.75)	Maternal age at delivery, calendar year at delivery, birth hospital, infant's sex, parity, maternal underlying medical conditions (eg, hypertension, psychiatric disorders, epilepsy, gestational diabetes and
		SGA	cOR 1.84 (1.33- 2.54)	aOR 1.88 (1.36-2.60)	

					preexisting diabetes), and socioeconomic status
30. Watson et al. 2017	AN (n=409 women)	Instrumental birth	Not reported	aRR 0.81 (0.59-1.11)	Maternal characteristics of household income, marital status, education, smoking during pregnancy, parity, and age
		C-section	Not reported	aRR 1.52 (1.11-2.10)	
		PTB	Not reported	aRR 1.39 (0.93-2.07)	
		SGA	Not reported	aRR 1.54 (1.09-2.17)	
31. Witt et al. 2012	Poor mental health before pregnancy (n=143 women)	LBW	cOR 2.39 (1.27-4.49)	aOR 1.99 (1.00-3.98)	Maternal age, race/ethnicity, marital status, education, health insurance, income, and number of children in the household
32. Wolgast et al. 2021	MDD diagnosis and used antidepressant medication one year before they became pregnant but discontinued ("before pregnancy group") (n=5,652 women)	Instrumental birth, planned C-section, unplanned C-section, overall C-section	*Instrumental birth: cOR 0.96 (0.86-1.06), planned c-section: cOR 1.31 (1.19-1.44), unplanned c-section: cOR 1.26 (1.15-1.37), overall c-section: cOR 1.28 (1.20-1.37)*	N/A	N/A
		PTB	cOR 1.17 (1.04-1.31)	aOR 1.12 (0.99-1.26)	Maternal age and childbirth, BMI, tobacco use during pregnancy, ART, diabetes, and other chronic diseases
		LBW	cOR 1.21 (1.06-1.39)	aOR 1.15 (1.00-1.33)	
		SGA	cOR 1.06 (0.88-1.27)	aOR 0.94 (0.78-1.15)	
		NNU admission	cOR 1.53 (1.20-1.96)	aOR 1.51 (1.17-1.95)	
33. Yedid Sion et al. 2016	Depression (n=221)	C-section	*cOR 2.48 (1.82-3.34)*	N/A	N/A
		PTB	*cOR 2.78 (1.52-5.09)*	N/A	N/A
		LBW	*cOR 1.95 (1.29-2.84)*	N/A	N/A

Abbreviations:
AN = Anorexia nervosa
BN = Bulimia nervosa
EDNOS = Eating disorders not otherwise specified
LBW = Low birthweight
PTB = Preterm birth
SGA = Small for gestational age
*Note: italicized values were calculated by hand, as only raw numbers were provided within the study 6.

6. Table S3: NOS quality assessment table for cohort studies and JBI Critical Appraisal Checklist for cross-sectional studies

Cohort Studies	SELECTION					COMPARABILITY	OUTCOME		
🟢 = Yes<br>🔴 = No<br>🟡 = Unclear<br>⚪ = Not applicable	1) Is the exposed cohort representative of the general population?	2) Is the exposed cohort representative of the study target population?	3) Was the non-exposed group selected from the same cohort as the exposed group?	4) Exposure status of all cases ascertained through validated methods?	5) Temporality: explicit that mental health problem predates pregnancy and birth outcomes?	1) Does the study control or adjust for relevant confounders?	1) Are birth outcomes ascertained using validated methods?	2) Was follow-up long enough for outcomes to occur?	3) Is there adequate follow up of study participants?
Aliaga et al. 2019	🔴	🔴	🟢	🟢	🟢	🟢	🟢	🟢	🔴
Ante et al. 2020	🟢	🟢	🟢	🟢	🟢	🟢	🟢	🟢	🟢
Bua et al. 2024	🔴	🔴	🟢	🟢	🟢	🟢	🔴	🟢	🟢
Bulik et al. 2009	🔴	🔴	🟢	🟢	🟢	🟢	🟢	🟢	🟡
Chatwin et al. 2025	🟢	🟢	🟢	🟢	🟢	🟢	🟢	🟢	🟢
Corti et al. 2019	🟡	🟡	🟢	🟢	🟢	⚪	🟢	🟢	🟢
Dadi et al. 2024	🔴	🔴	🟢	🟢	🟢	🟢	🟢	🟢	🟢
Eagles et al. 2012	🟢	🟢	🟢	🟢	🟢	🟢	🟢	🟢	🟢
Gavin et al. 2009	🟡	🟢	🟢	🟢	🟢	⚪	🟢	🟢	🟢
Haas et al. 2005	🟡	🔴	🟢	🟢	🟢	🟢	🟢	🟢	🔴
Jensen et al. 2013	🟢	🟢	🟢	🟢	🟢	🟢	🟢	🟢	🟢
Kang-Yi et al. 2018	🔴	🟡	🟢	🟢	🟢	🟢	🟢	🟢	🟡
Kouba et al. 2005	🔴	🔴	🟢	🟢	🟢	⚪	🟢	🟢	🟡
Langham et al. 2023	🟢	🟢	🟢	🟢	🟢	🟢	🟢	🟢	🟢
Mantel et al. 2020	🟢	🟢	🟢	🟢	🟢	🟢	🟢	🟢	🟢
Mei-Dan et al. 2015	🟢	🟡	🟢	🟢	🟢	🟢	🟢	🟢	🟡
Micali et al. 2016	🟢	🔴	🟢	🟢	🟢	🟢	🟢	🟢	🔴
Momen et al. 2025	🟢	🟢	🟢	🟢	🟢	🟢	🟢	🟢	🟢
Phillips et al. 2010	🔴	🔴	🟢	🟢	🟢	🟢	🔴	🟢	🔴
Shaw et al. 2014	🔴	🟢	🟢	🟢	🟢	🟢	🟢	🟢	🟢
Sollid et al. 2004	🟢	🟢	🟢	🟢	🟢	🟢	🟢	🟢	🟢
Spry et al. 2020	🟢	🟡	🟢	🟢	🟢	🟢	🟡	🟢	🔴
Vigod et al. 2014	🟢	🟢	🟢	🟢	🟢	🟢	🟢	🟢	🟢
Vigod et al. 2020	🟢	🟢	🟢	🟢	🟢	🟢	🟢	🟢	🟢
Wang et al. 2021	🔴	🟢	🟢	🟢	🟢	🟢	🟢	🟢	🟢
Watson et al. 2017	🔴	🔴	🟢	🟢	🟢	🟢	🟢	🟢	🟡
Wolgast et al. 2021	🟢	🟢	🟢	🟢	🟢	🟢	🟢	🟢	🟢
YedidSion et al. 2016	🔴	🟢	🟢	🟢	🟢	⚪	🟢	🟢	🟡

**JBI - Cross Sectional**	1) Were the criteria for inclusion in the sample clearly defined?	2) Were the study subjects and the setting described in detail?	3) Was the exposure measured in a valid and reliable way?	4) Were objective, standard criteria used for measuring the condition?	5) Were confounding factors identified?	6) Were strategies to deal with confounding factors stated?	7) Were the outcomes measured in a valid and reliable way?	8) Was appropriate statistical analysis used?	9) Is the study sample representative of the study target population?	10) Is the study sample group representative of the general population?
Ciesielski et al. 2015	red	green	green	green	green	green	green	green	yellow	yellow
Latendresse et al. 2015	green	green	green	green	yellow	yellow	green	green	red	red
Mongan et al. 2019	green	green	green	green	green	green	green	green	green	green
Raisanen et al. 2014	green	green	green	green	grey	grey	green	grey	green	green
Witt et al. 2012	green	yellow	green	green	green	green	green	green	green	green

7. **Pooled crude and adjusted associations between any pre-existing mental health problems and instrumental birth**

   a. **Pooled crude OR/RR values for instrumental birth**

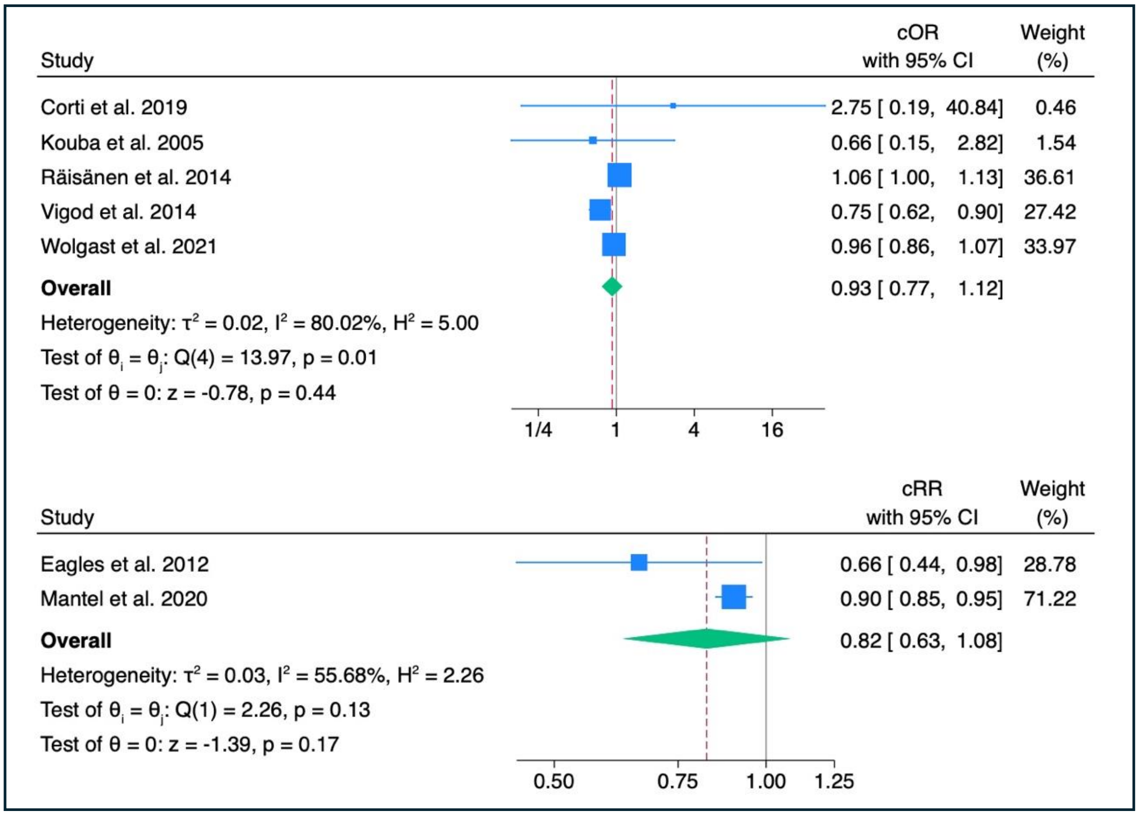

   b. **Pooled adjusted OR/RR values for instrumental birth**

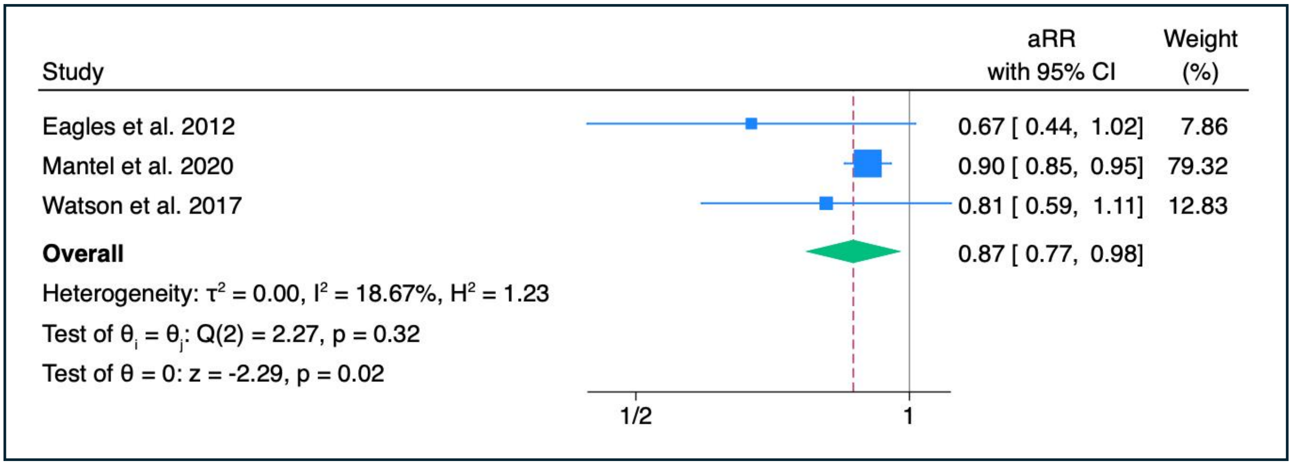

8. **Pooled adjusted associations between any pre-existing mental health problems and caesarean section**

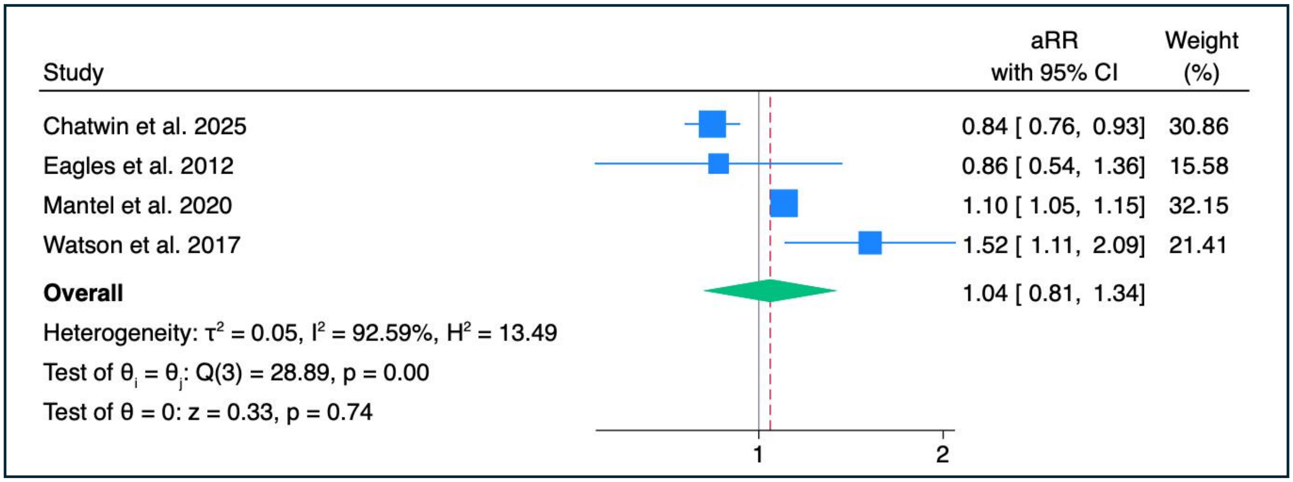

*Watson et al. 2017 was included instead of Bulik et al. 2009

9. **Crude associations between any pre-existing mental health problems and planned c-section**

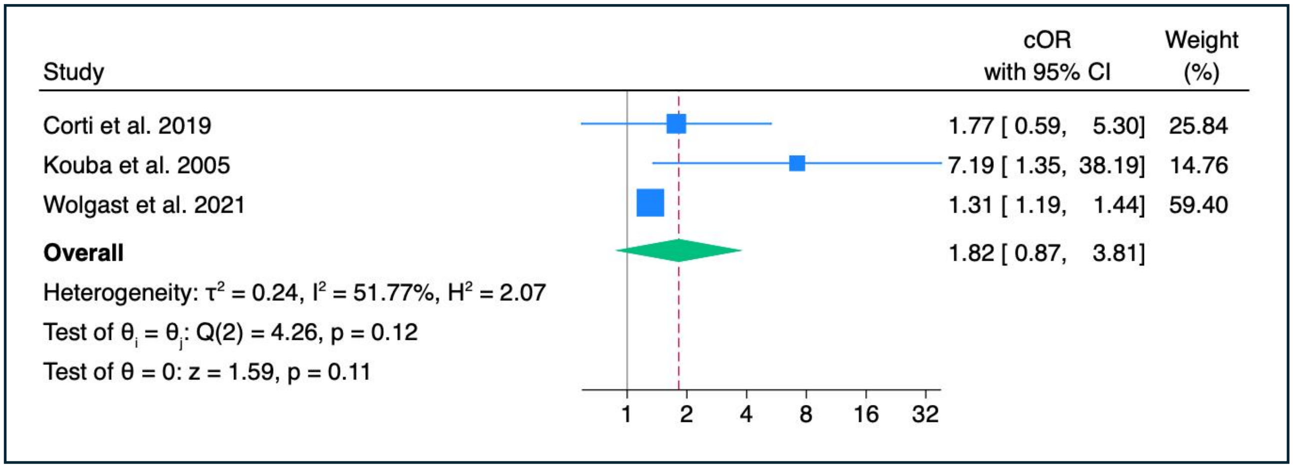

10. **Crude associations between any pre-existing mental health problems and unplanned c-section**

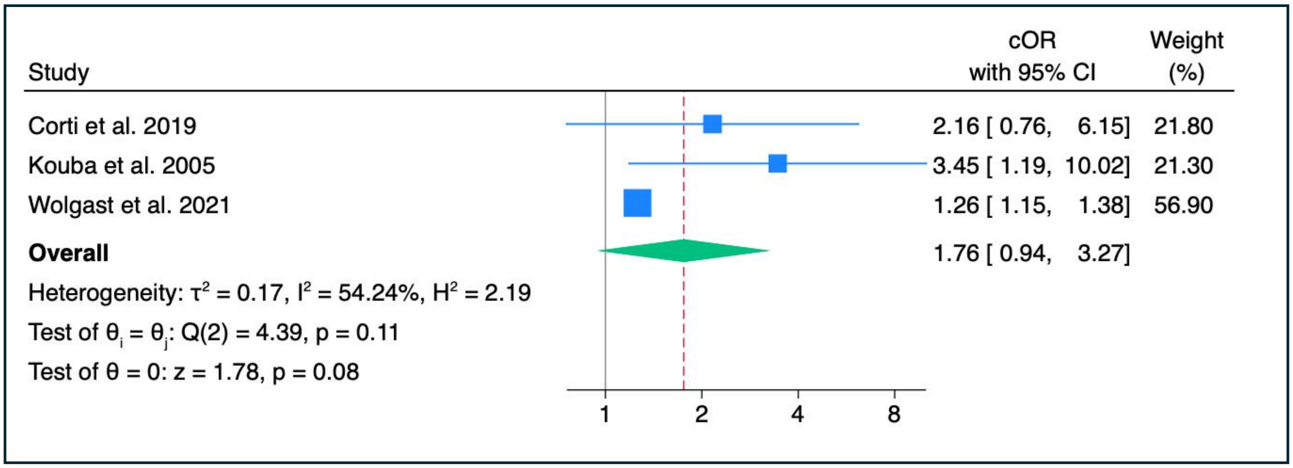

11. **Funnel plot for pooled crude associations between any pre-existing mental health problems and PTB**

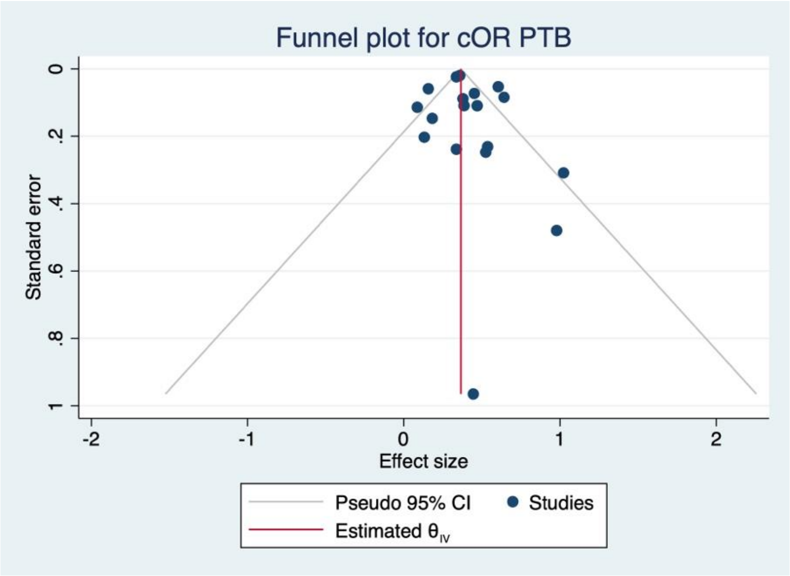

12. Pooled adjusted OR/RR between any pre-existing mental health problems and PTB

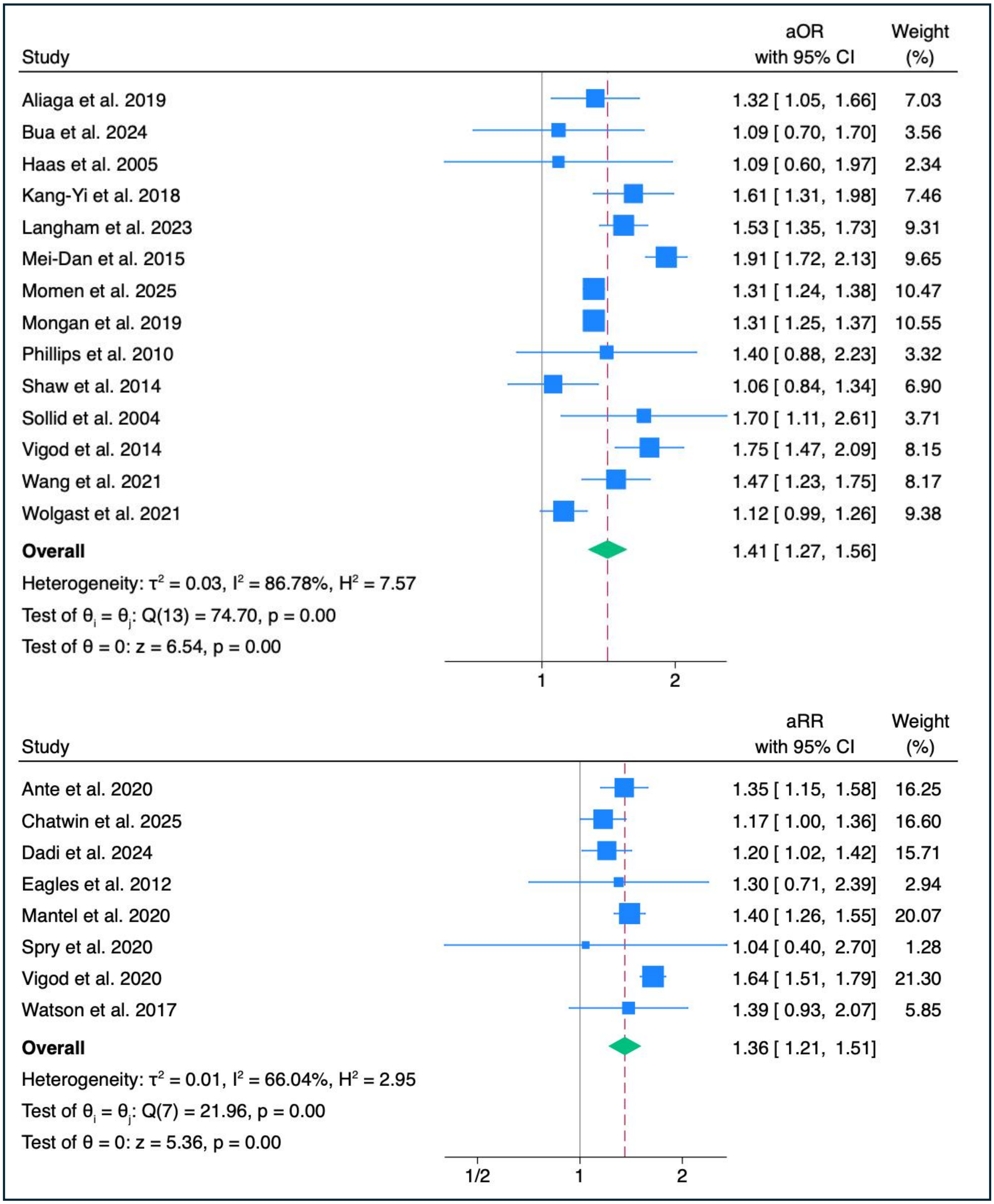

13. **Funnel plot for pooled adjusted associations between any pre-existing mental health problems and PTB**

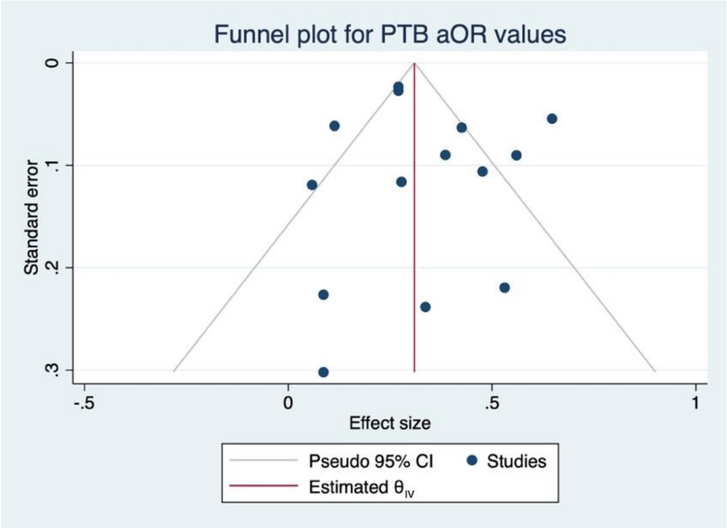

14. **Pooled adjusted OR/RR between any pre-existing mental health problems and LBW**

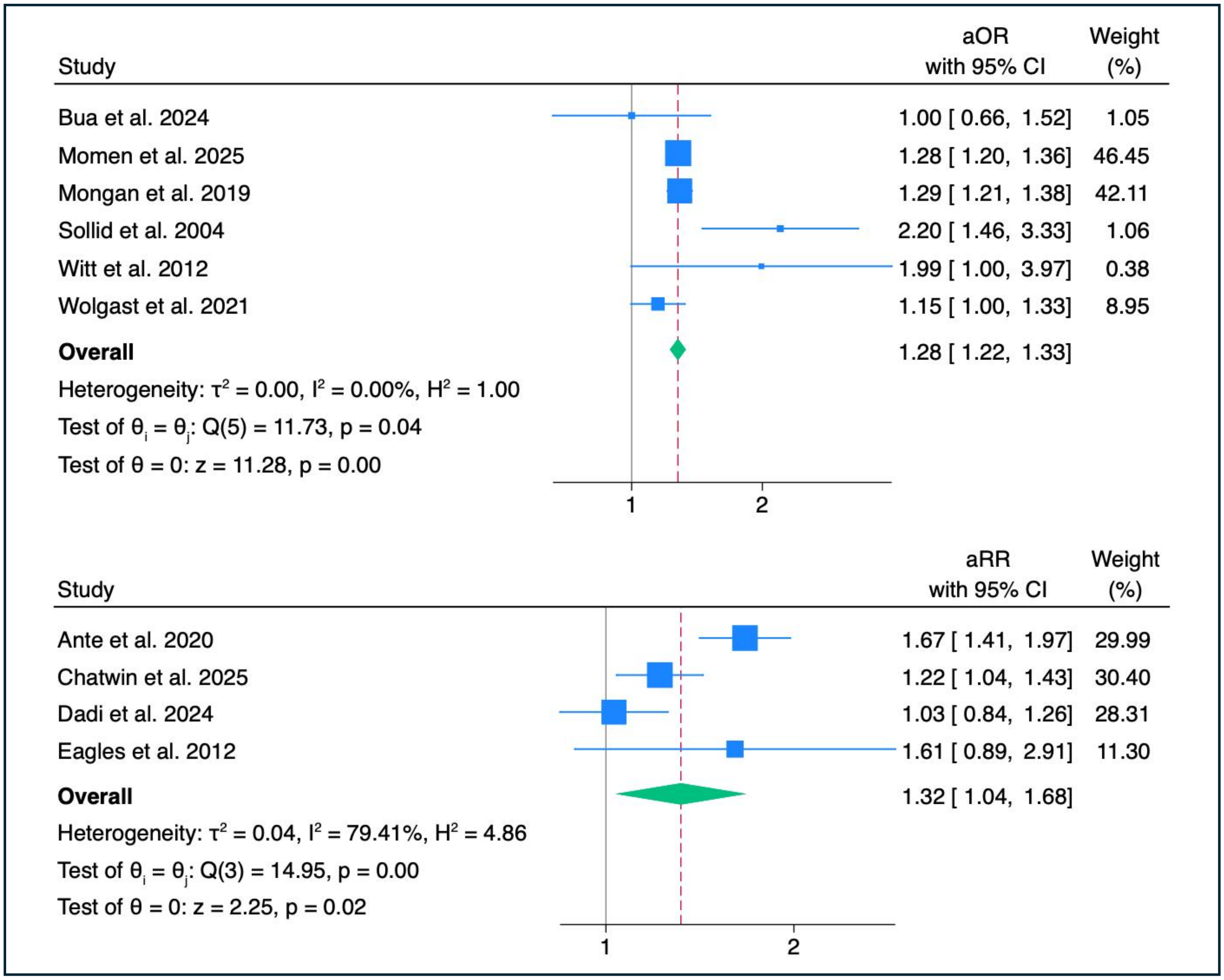

15. Pooled adjusted OR/RR between any pre-existing mental health problems and SGA

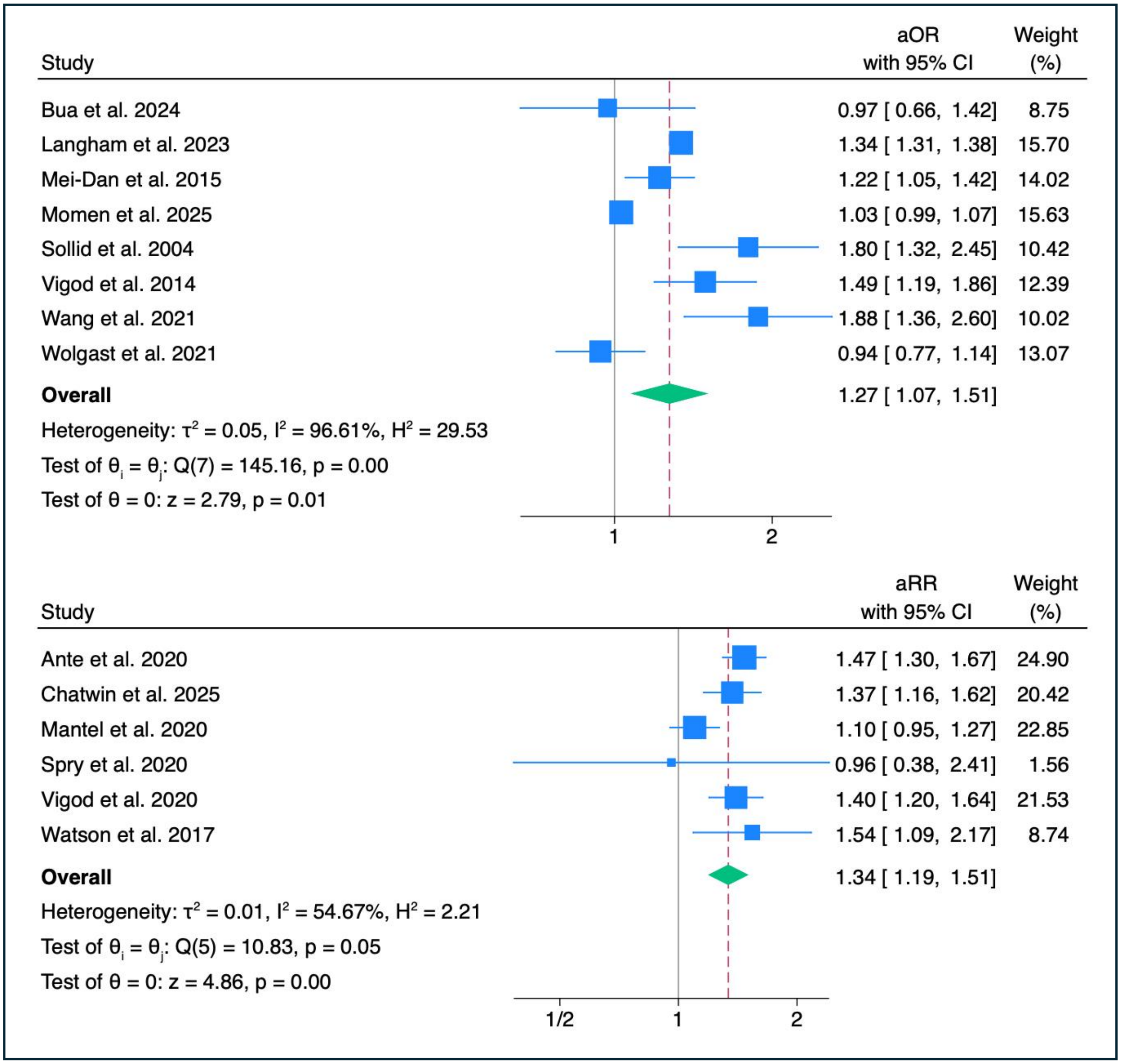

16. **Pooled crude and adjusted ORs between any pre-existing mental health problems and NNU admission**

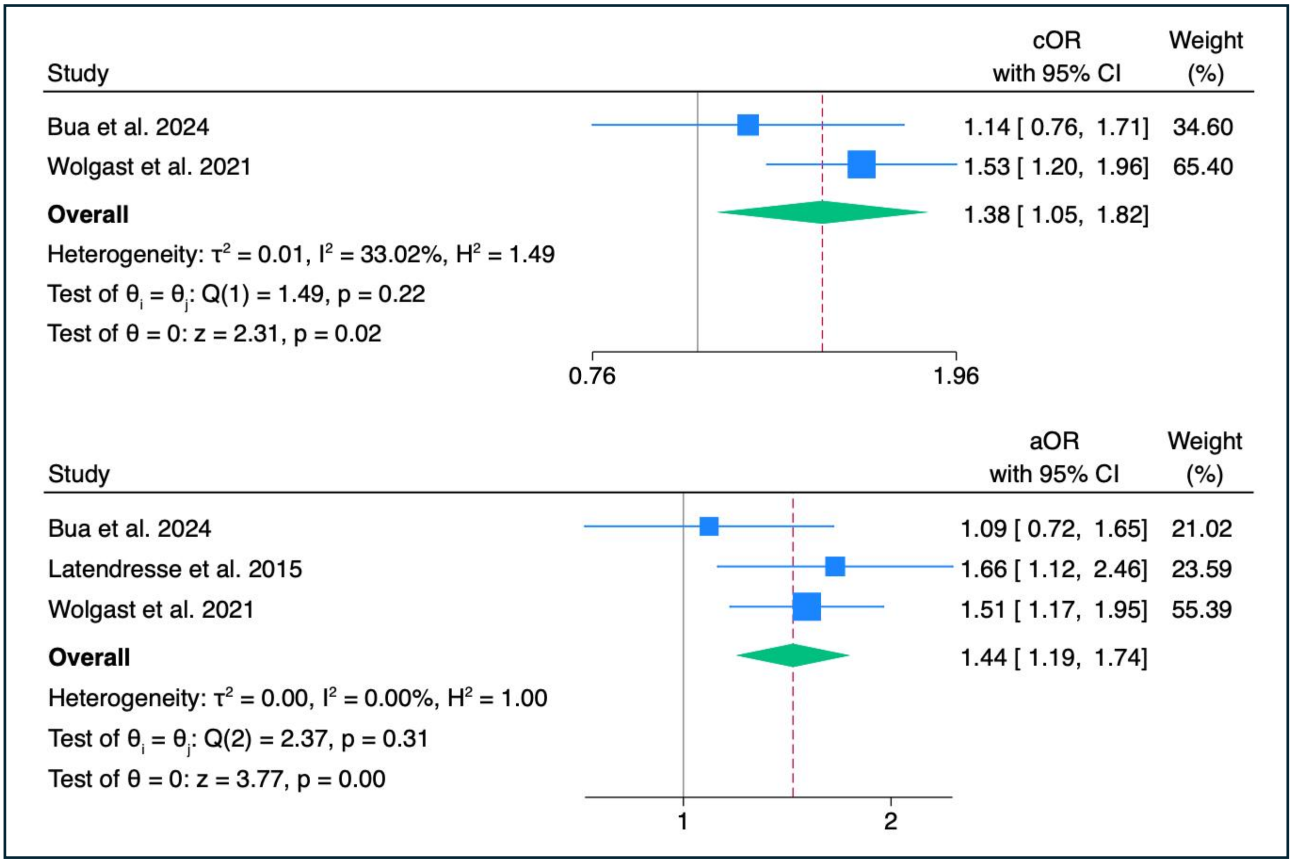

17. Table S4: Summary table for sensitivity analyses of crude analyses of birth outcomes following meta-analysis (higher quality studies)

	**All studies**						**High quality studies only**					
**Outcome**	***n***	***cOR (95% CI)***	***I^2***	***n***	***cRR (95% CI)***	***I^2***	***n***	***cOR (95% CI)***	***I^2***	***n***	***cRR (95% CI)***	***I^2***
**1.Mode of birth**												
**1.1 Instrumental birth** (v normal vaginal birth)	5	0.93 (0.77, 1.12)	80.0	2	0.82 (0.63, 1.08)	55.7	2	0.86 (0.68, 1.09)	80.5	2	0.82 (0.63, 1.08)	55.7
**1.2 Caesarean section**[a] (v normal vaginal birth)	7	**1.53 (1.20, 1.97)**	98.4	5	**1.09 (1.06, 1.13)**	0	3	**1.33 (1.28, 1.39)**	33.6	4	**1.09 (1.06, 1.13)**	0
**2.Preterm birth** (v term birth)	18	**1.48 (1.35, 1.62)**	83.8	8	**1.39 (1.25, 1.54)**	59.9	6	**1.48 (1.29, 1.69)**	93.0	5	**1.43 (1.27, 1.60)**	69.3
**3.Low birthweight** (v normal birthweight)	9	**1.48 (1.31, 1.67)**	78.7	4	**1.40 (1.13, 1.75)**	77.6	5	**1.45 (1.30, 1.62)**	82.3	3	**1.53 (1.29, 1.80)**	47.7
**4.Small for gestational age** (v not SGA)	10	**1.37 (1.13, 1.66)**	96.7	6	**1.29 (1.15, 1.44)**	50.3	5	**1.38 (1.06, 1.80)**	98.6	4	**1.29 (1.15, 1.45)**	63.9
**5. NNU admission** (v no NNU admission)	2	**1.38 (1.05, 1.82)**	33.0	0	-	-	1[b]	**1.53 (1.20-1.96)**	-	0	-	-

[a]No high-quality studies reported on planned or unplanned caesarean section

[b]Effect sizes from individual studies are reported as meta-analysis is not possible

18. Table S5: Summary table for sensitivity analyses of crude analyses of birth outcomes following meta-analysis (common mental health problems, severe mental health problems, and eating disorders compared to original analysis including all studies)

	All studies						Common mental health problems only						Severe mental health problems only						Eating disorders only					
**Outcome**	***n***	***cOR***	I^2	***n***	***cRR***	I^2	***n***	***cOR***	I^2	***n***	***cRR***	I^2	***n***	***cOR***	I^2	***n***	***cRR***	I^2	***n***	***cOR***	I^2	***n***	***cRR***	I^2
**1.Mode of birth**																								
**1.1 Instrumental birth** (v normal vaginal birth)	5	0.93 (0.77, 1.12)	80.0	2	0.82 (0.63, 1.08)	55.7	3	1.02 (0.93, 1.12)	43.6	0	-	-	1[a]	**0.75 (0.62-0.90)**	-	0	-	-	1[a]	0.66 (0.14, 2.55)	-	2	0.82 (0.63, 1.08)	55.7
**1.2 Caesarean section** (v normal vaginal birth)	7	**1.53 (1.20, 1.97)**	98.4	5	**1.09 (1.06, 1.13)**	0	5	**1.48 (1.11, 1.96)**	98.8	0	-	-	2	**1.35 (1.25, 1.45)**	0	1[a]	**1.09 (1.04, 1.14)**	-	2	1.95 (0.49, 7.82)	88.5	4	**1.09 (1.05, 1.14)**	0
**2.Preterm birth** (v term birth)	18	**1.48 (1.35, 1.62)**	83.8	8	**1.39 (1.25, 1.54)**	59.9	11	**1.36 (1.18, 1.57)**	82.5	1[a]	1.02 (0.42, 2.49)	-	4	**1.70 (1.48, 1.95)**	68.6	1[a]	**1.65 (1.51-1.79)**	-	3	**1.49 (1.07, 2.08)**	49.9	5	**1.35 (1.26, 1.45)**	0
**3.Low birthweight** (v normal birthweight)	9	**1.48 (1.31, 1.67)**	78.7	4	**1.40 (1.13, 1.75)**	77.6	5	**1.37 (1.15, 1.64)**	68.9	0	-	-	2	**1.49 (1.28, 1.73)**	49.6	0	-	-	2	1.58 (0.93, 2.67)	83.5	3	**1.53 (1.29, 1.80)**	47.7
**4.Small for gestational age** (v not SGA)	10	**1.37 (1.13, 1.66)**	96.7	6	**1.29 (1.15, 1.44)**	50.3	4	**1.16 (1.12, 1.20)**	0	1[a]	1.08 (0.42, 2.76)	-	4	**1.39 (1.17, 1.65)**	64.2	1[a]	**1.44 (1.23-1.69)**	-	3	1.98 (0.88, 4.47)	93.4	4	**1.25 (1.10, 1.43)**	55.8
**5. NNU admission** (v no NNU admission)	2	**1.38 (1.05, 1.82)**	33.0	0	-	-	2	**1.38 (1.05, 1.82)**	33.0	0	-	-	0	-	-	0	-	-	0	-		0	-	-

[a]Effect sizes from individual studies are reported as meta-analysis is not possible

19. Table S6: Summary table for sensitivity analysis of articles with no comorbidity in comparison groups and possible comorbidity, compared to the main analysis including all studies

	All studies						No comorbidity in comparison group						Possible comorbidity in comparison group					
**Outcome**	***n***	***cOR***	I^2	***n***	***cRR***	I^2	***n***	***cOR***	I^2	***n***	***cRR***	I^2	***n***	***cOR***	I^2	***n***	***cRR***	I^2
**1.Mode of birth**																		
**1.1 Instrumental birth** (v normal vaginal birth)	5	0.93 (0.77, 1.12)	80.0	2	0.82 (0.63, 1.08)	55.7	2	**0.75 (0.63, 0.91)**	0	1[a]	0.66 (0.44-0.98)	-	3	1.02 (0.92, 1.12)	44.4	1[a]	**0.66 (0.44, 0.98)**	-
**1.2 Caesarean section[b]** (v normal vaginal birth)	7	**1.53 (1.20, 1.97)**	98.4	5[c]	**1.09 (1.06, 1.13)**	0	3	**1.36 (1.32, 1.40)**	0	2	1.04 (0.84, 1.27)	35.2	3	1.58 (0.83, 3.03)	99.4	2	0.80 (0.54, 1.19)	0
**2.Preterm birth** (v term birth)	18	**1.48 (1.35, 1.62)**	83.8	8	**1.39 (1.25, 1.54)**	59.9	9	**1.58 (1.44, 1.73)**	82.8	3	**1.46 (1.17, 1.81)**	72.3	7	**1.29 (1.14, 1.47)**	24.8	2	0.93 (0.41, 2.09)	0
**3.Low birthweight** (v normal birthweight)	9	**1.48 (1.31, 1.67)**	78.7	4	**1.40 (1.13, 1.75)**	77.6	6	**1.52 (1.41, 1.64)**	45.2	2	1.34 (0.78, 2.32)	73.4	2	**1.19 (1.05, 1.36)**	0	3	**1.53 (1.29, 1.80)**	47.7
**4.Small for gestational age** (v not SGA)	10	**1.37 (1.13, 1.66)**	96.7	6	**1.29 (1.15, 1.44)**	50.3	6	**1.47 (1.18, 1.82)**	97.7	1[a]	**1.44 (1.23-1.69)**	-	3	1.05 (0.89, 1.24)	0	2	1.09 (0.53, 2.22)	0
**5. NNU admission** (v no NNU admission)	2	**1.38 (1.05, 1.82)**	33.0	0	-	-	0	-	-	0	-	-	0	-	-	0	-	-

[a]Effect sizes from individual studies are reported as meta-analysis is not possible

20. Management of potential data source overlap

For transparency, tables below outline any potential overlaps in data sources, along with explanations for how this was evaluated and addressed for each pooled analysis:

Authors, Year	Country	Time frame + Population	Dataset used	Outcomes reported	OR/RR
Chatwin et al. 2025	Denmark (national)	The study cohort included all liveborn singletons in Denmark between 1991 and 2015	Danish Medical Birth	PTB, LBW, SGA	RR
Sollid et al. 2004	Denmark (national)	Women who were hospitalized with an eating disorder as the primary psychiatric diagnosis followed by a registered birth of a single child during 1973 to 1993	Danish Medical Birth	PTB, LBW, SGA	OR
Micali et al. 2016	Denmark (national)	Danish national birth cohort (100 418 pregnancies recruited from 1996 to 2002 at the first antenatal visit throughout Denmark). Eligibility for this specific study required that the women had participated in the initial telephone interview carried out at 16–17 weeks' gestation	Danish national birth cohort	SGA	OR
Momen et al. 2025	Denmark (national)	Children of mothers with a previous but not recent mental disorder diagnosis (i.e. more than 2 years prior to conception of index child) in the Central Psychiatric Register Liveborn singletons during 1997–2015 in Denmark	Danish Medical Birth	C-section, PTB, LBW, SGA	OR
Jensen et al. 2013	Denmark (national)	Data on all pregnancies from 1996 to 2006 were obtained	Danish Medical Birth	SGA	HR

Jensen et al. 2013 reported hazard ratios therefore was not pooled with other Danish studies, only described descriptively. Chatwin et al. 2025 was only the study reporting RRs, therefore was never pooled with other Danish studies which reported ORs. For Sollid et al. 2004 and Momen et al. 2025, although both studies use Danish register data and include women with eating disorders diagnosed before pregnancy, the birth periods do not overlap (1973–1993 vs 1997–2015). Therefore, these two studies were included in the same meta-analyses. For Sollid et al. 2004 and Micali et al. 2016, birth periods also do not overlap (1973-1993 vs 1996-2002). For Momen et al. (2025) and Micali et al. (2016), there is potential overlap in pregnancies, as both include Danish births occurring between 1997 and 2002, with the Danish National Birth Cohort nested within the national birth population. Therefore, these two studies were not pooled in the same analyses; Momen et al. (2025) was prioritised due to its larger and more recent sample.

Authors, Year	Country	Time frame + Population	Dataset used	Outcomes reported	OR/RR
Kouba et al. 2005	Sweden (Stockholm)	Screening interviews were conducted between August 1997 and June 2001, women were in early pregnancy (gestational week 10)	Recruitment from 13 prenatal clinics in Stockholm	Instrumental birth, planned c-section, unplanned c-section, c-section overall, PTB, SGA	OR
Mantel et al. 2020	Sweden (national)	All singleton births included in the Swedish Medical Birth Register from January 1, 2003, to December 31, 2014	Swedish Medical Birth Register	Instrumental birth, C-section overall, PTB, SGA	RR
Wolgast et al. 2021	Sweden (national)	All women born in Sweden between 1973 and 1993, who gave birth during the years 2012–2015 (n = 262 329). Only first born child included.	Swedish medical birth register	Instrumental birth, planned C-section, unplanned C-section, overall C-section, PTB, LBW, SGA	OR

Mantel et al. (2020) and Wolgast et al. (2021) both used the Swedish Medical Birth Register and included births occurring between 2012 and 2014. However, these studies reported different effects (RRs vs ORs) therefore were never pooled in the same meta-analysis. Kouba et al. 2005 and Wolgast et al. 2021 both reported ORs but birth periods do not overlap.

Authors, Year	Country	Time frame + Population	Dataset used	Outcomes reported	OR/RR
Bulik et al. 2009	Norway (national)	The present study is based on the first and fourth questionnaires, based on version 3 of the quality-assured data files released in 2007	Norwegian Mother and Child Cohort study	C-section overall, PTB, SGA	RR
Watson et al. 2017	Norway (national)	Current study is based on Version 8 of the quality-assured data files released for research in 2015	Norwegian Mother and Child Cohort study	Instrumental birth, C-section overall, PTB, SGA	RR

Bulik et al. 2009 and Watson et al. 2017 were both based on data from the Norwegian Mother and Child Cohort Study (MoBa), creating potential overlap in study participants. To avoid double counting, Watson et al. 2017 was prioritised in pooled meta-analyses because it included a larger and more recent sample. However, for outcomes where Watson et al. 2017 did not report the required crude effect estimates, Bulik et al. 2009 was included instead. Under no circumstances were both studies included in the same pooled analysis.
