# Supplementary material for: Birth outcomes for women with pre-existing mental health problems: a systematic review and meta-analysis
Source: BMJ Open. 2026 May 29;16(5):e106566. doi: 10.1136/bmjopen-2025-106566 (PMC13223673; doi:10.1136/bmjopen-2025-106566)
Supplement: Supplementary file 2 [file bmjopen-2025-106566.reviewer_comments.pdf]

PEER REVIEW HISTORY

BMJ Open publishes all reviews undertaken for accepted manuscripts. Reviewers are asked to complete a checklist review form and are provided with free text boxes to elaborate on their assessment. These free text comments are reproduced below.

ARTICLE DETAILS

Title (Provisional)

Birth outcomes for women with pre-existing mental health problems: a systematic review and meta-analysis

Authors

Gong, Jenny; Henderson, Ian; Lynch, Rosie; Daskalopoulou, Zoe; Roberts, Nia; Fellmeth, Gracia; Harrison, Sian; Quigley, Maria; Alderdice, Fiona

---

VERSION 1 - REVIEW

**Reviewer**	**1**
**Name**	**González, José Antonio**
**Affiliation Department**	**Barcelona-Tech, Statistics and Operations Research**
**Date**	**09-Sep-2025**
**COI**	**None**

Birth outcomes for women with pre-existing mental health problems: a systematic review and meta-analysis

Manuscript ID bmjopen-2025-106566

The authors address in their paper the study of relationships between pre-existing mental health problems in women and different issues in their deliveries. They argue that there is a gap in the literature, which has focused in problems appearing during pregnancy, while it seems relevant to incorporate new knowledge related to mental health problems in future mothers in order to take measures to reduce risks for the new-born children. The methodology employed is a systematic review and meta-analysis, according to the reporting guidelines proposed in the PRISMA recommendations.

I have no major concerns with the manuscript, the structure is clear and easy to follow. I have just one important issue to emphasise, since I think that it has been completely left aside in the paper, and readers deserve to find this point included in the synthesis.

All the studies for this matter are obviously observational. PRISMA was mainly addressed for systematic reviews of randomized controlled trials in order to increase accuracy in

estimates for specific questions, among other objectives, like dealing with heterogeneity and exploring possible explanations of clinical nature. PRISMA was not closed to other types of studies, but there are abundant research pointing out that observational studies are prone to bias their results even if adjustment for covariates is taken into account.

In this paper, I've missed a sufficient degree of caution on the part of the authors to highlight this limitation in their research. It's true that we can see in the subsection "Strengths and limitations" that residual confounding is still possible, but this affirmation seems devoid of context.

The Table 1 shows the Study Design for each study. The reader can find many types: retrospective cohort (study), prospective cohort, cohort, cohort (within some period), cross-sectional, nested case control, Population based cohort. In my opinion, some of these types could be unified, since it's clear that the different authors had qualified their respective works according to their own codes, especially in the case of the cohorts, which appear to be mainly based in historical records. Not only in my opinion, also in CONSORT's opinion, the "prospective" and "retrospective" names are often confusing and depend on various meanings, so it's advisable not to use them. Besides, it's commonly accepted that "prospective" cohorts are a type of design that can contribute with more "evidence" than "retrospective" cohorts. Unfortunately, there is not an easy way to consider this aspect in the analysis, and we observe that the "retrospective" ones, with larger sample sizes, have also notable larger weight. Just for only this reason, I would enhance the caution in the interpretation of the results.

In the Abstract, I would suggest to moderate the tone in two sentences. First, instead of "To synthesize evidence examining the impact of mental health …", I'd try to use another word to replace "impact", which refers to me to a direct causal effect of the exposure, perhaps something like "association". And second, "… studies reported different measures of effect"; for the same reason, I'd prefer not to listen of measures of effect here.

Back to the Discussion section, which is very important to me since it's the place where the authors can qualify their findings, which may seem too impressive in regard to the numbers appearing as results. As many meta-analyses were obtained according to the type of mental problem and measure of association, I ask the authors do not forget to remind the reader that not all analyses include a high number of studies. The starting sentence "This systematic review and meta-analysis examined 33 studies…" suggests an eminent and powerful analysis, which is not exactly like that. On the contrary: the multiplicity of analysis is bringing down the strength of the conclusions, which is another argument to increase caution in the interpretation.

The Summary includes increases in odds and risks for the different pathologies. I miss some measure of uncertainty, even if there is reasonable doubt about the accuracy of these results. I am also missing a brief reference to the detected heterogeneity in almost each analysis. The topic is commented along the document, regarding different definitions of exposure, covariates, timings and other conditions that could introduce further variability,

but may be worth to recall it again in this Summary.

By the way, the point estimates don't match when I compare them. For instance, "PTB (39% increased odds or 36% increased risk)", but Figure 3 shows cOR 1.48 and cRR 1.39. And so on with LBW, SGA and NNU. Perhaps this should be clarified.

The authors say that the countries included were the so-called "high-income" by the World Bank, but this set of countries is slightly different every year so the paper should indicate the year of reference. For instance, Russia has been excluded in the search although it was classified as "high-income country" in 2024. The update of the search could had taken this and maybe other changes into account.

Another confusion arises from this note at foot of Table 1: "Bulik et al. 2009 and Watson et al. 2017 both used the Norwegian Mother and Child Cohort Study (MoBa), therefore only the larger and more recent study (Watson et al. 2017) was included in meta-analyses to avoid double counting participants". However, we can clearly see the study from Bulik et al at different meta-analyses: Figure 2 (cRR); Figure 3 (cRR); Figure 5 (cRR), but the name of Watson is appearing nowhere. Please clarify.

The assessment of the risk of bias is a key topic in any systematic review. However, the instruments employed here, initially suitable for observational studies, are substantially weak, when compared with similar instruments applied to randomized controlled trials. Table 2 shows the assessments obtained with the NOS and JBI tools. Leaving aside the difficulty to answer questions like the representativeness of the cohort, or why some topics are always answered with "Yes", I see that this method has a serious drawback: it may give the impression that a study with many positive answers is free of bias or even is as good as a RCT. Fortunately, the assessment is only used to "validate" the analysis with a subgroup of "high-quality" studies.

The analysis of sensitivity performed is not conclusive, which is actually positive, as it does not reveal strong discrepancies. Nonetheless, the authors' interpretation feels somewhat subjective. They appear satisfied because the results from "high quality" studies remained similar to the whole set. However, when the type of mental health problem is taken into account, they identify changes worth noting —although such claims remain vague due to the wide confidence intervals. Maybe, a chart would more useful for this purpose instead of a table, and the interpretation would appear objectively, with not as many words.

**Reviewer**	**2**
**Name**	**Nesheim, BrittIngjerd**
**Affiliation**	**University of Oslo, Faculty of Medicine**
**Date**	**15-Sep-2025**
**COI**	**None**

This is a well performed metaanalysis, addressing an important question. It is well written, clear and understandable. The references are adequate. I am supportive of the publication of this paper.

**Reviewer**	**3**
**Name**	**Singh, Megha**
**Affiliation**	**University of Birmingham, Applied health research**
**Date**	**27-Jan-2026**
**COI**	**None**

Overall

The exposure “pre-existing mental health problems” is not clearly defined. It is unclear whether these conditions refers to active illness or history, and how very different disorders are combined into one group, contributing to heterogeneity and difficulty in interpreting the findings. Throughout the paper, findings are frequently compared with evidence on antenatal or perinatal mental health and pregnancy outcomes. It is important to recognise that, although pre-existing mental health conditions may be associated with mental health during pregnancy, these represent distinct exposures and therefore different research questions that should be clearly separated in interpretation.

Abstract

- What mental health problems prior to pregnancy, please clarify.
- What was the period of search
- Rather than repetition of exposure and outcomes in abstract the abstract could benefit from more information on methodology, what was the design of studies included, if more than one reviewer involve in screening and data extraction, Also what checklist used for quality assessment of studies.
- Results do not specify how many studies were identified per outcome.
- Conclusion suggest “preventing the onset or recurrence of mental health problems during the perinatal period” which is out of scope of this study.

Strengths and Limitations

Contradictory claim made that exposure definition was preset as a strength and this is also mentioned as limitation, this require clarification.

Introduction

- Introduction focuses heavily on mental health during pregnancy, not pre-existing mental health before pregnancy, which is the actual exposure of this review.
- Introduction drift between pre-existing, antenatal, and perinatal mental health without clearly separating these.
- Over-reliance on literature about antenatal depression/anxiety and antidepressant exposure.
- Excessive detail on long-term consequences of PTB/LBW/SGA, which is not directly needed to justify the research question.

- Limited explanation of why pre-existing mental health problems could independently affect birth outcomes.
- Knowledge gap is stated broadly but not clearly demonstrated in contrast to existing reviews.
- Exposure definition (pre-existing mental health) is not clearly unpacked (active vs past history, chronic vs episodic, treated vs untreated).

Methods

- The exposure definition is not clearly specified at the outset. Although substance use disorders were excluded, the scope of included mental health conditions remains unclear, Broad search terms (stress, mania, PTSD) further suggest the absence of a tightly defined exposure framework. Moreover, some studies included had relied on self-reported symptoms rather than clinical diagnoses.
- A large number of studies were excluded due to unclear timing of exposure, yet several included studies report "unspecified pre-existing mental health problems."
- Highly heterogeneous conditions (depression symptoms, clinical diagnoses, anxiety, PTSD, schizophrenia, eating disorders, affective disorders) are synthesised under a single category without justification.
- Study design heterogeneity (cohort and cross-sectional studies) and substantial variation in covariate adjustment are not accounted for in synthesis decisions, despite apparent pooling of adjusted estimates.
- The rationale for excluding antenatal and postnatal mental health outcomes is not explained.
- Discrepancies in the PRISMA flow diagram require verification.

Results

- Grouping exposures into clinically meaningful categories and presenting as main analysis with effect estimates would have been better
- Very high heterogeneity is reported but not explored or justified.
- The quality assessment section should describe the specific methodological weaknesses observed across studies, rather than only reporting overall scores

Discussion

- Comparisons with antenatal mental health meta-analyses imply similar mechanisms, without acknowledging that antenatal and pre-existing conditions are epidemiologically and clinically different exposures.

- The limitations suggest that differences between studies are mainly due to broad research questions and different populations, but they do not recognise that a major reason for this variation is combining very different mental health conditions into one group and pooling crude and adjusted results together.

VERSION 1 - AUTHOR RESPONSE

REVIEWER 1:

1. All the studies for this matter are obviously observational. PRISMA was mainly addressed for systematic reviews of randomized controlled trials in order to increase accuracy in estimates for specific questions, among other objectives, like dealing with heterogeneity and exploring possible explanations of clinical nature. PRISMA was

not closed to other types of studies, but there are abundant research pointing out that observational studies are prone to bias their results even if adjustment for covariates is taken into account.

We agree with the reviewer that observational studies are susceptible to bias, even after adjustment for confounders. In response to this comment, we strengthened the discussion of residual confounding and unmeasured bias as a key limitation of this review. We have also emphasised that variation in covariate selection and incomplete adjustment across studies may have influenced pooled estimates, and that associations should be interpreted cautiously in light of these limitations (see pg. 17 under strengths and limitations).

2. In this paper, I've missed a sufficient degree of caution on the part of the authors to highlight this limitation in their research. It's true that we can see in the subsection "Strengths and limitations" that *residual confounding is still possible*, but this affirmation seems devoid of context.

As addressed in our response to the previous comment, we have further strengthened the discussion of residual confounding to provide clearer context regarding its potential impact on the findings (see pg. 17 under strengths and limitations).

3. The Table 1 shows the *Study Design* for each study. The reader can find many types: retrospective cohort (study), prospective cohort, cohort, cohort (within some period), cross-sectional, nested case control, Population based cohort. In my opinion, some of these types could be unified, since it's clear that the different authors had qualified their respective works according to their own codes, especially in the case of the cohorts, which appear to be mainly based in historical records. Not only in my opinion, also in CONSORT's opinion, the "prospective" and "retrospective" names are often confusing and depend on various meanings, so it's advisable not to use them. Besides, it's commonly accepted that "prospective" cohorts are a type of design that can contribute with more "evidence" than "retrospective" cohorts. Unfortunately, there is not an easy way to consider this aspect in the analysis, and we observe that the "retrospective" ones, with larger sample sizes, have also notable larger weight. Just for only this reason, I would enhance the caution in the interpretation of the results.

We agree that terminology such as "prospective" and "retrospective" cohort can be inconsistently applied across observational studies. In response, we have revised the study characteristics table to simplify the classification of study design, now categorising studies as either cohort or cross-sectional. This change was made to improve clarity and avoid over-interpretation of design labels (see Table 1).

4. In the Abstract, I would suggest to moderate the tone in two sentences. First, instead of "To synthesize evidence examining the impact of mental health …", I'd try to use another word to replace "impact", which refers to me to a direct causal effect of the exposure, perhaps something like "association". And second, "… studies reported

different measures of effect"; for the same reason, I'd prefer not to listen of *measures of effect* here.

We have revised the Abstract to remove causal language, replacing "impact" with "association," and changed "measures of effect" to "measures of association" to better reflect the observational nature of the included studies. We have ensured that causal terminology has been removed throughout the manuscript.

5. Back to the Discussion section, which is very important to me since it's the place where the authors can qualify their findings, which may seem too impressive in regard to the numbers appearing as results. As many meta-analyses were obtained according to the type of mental problem and measure of association, I ask the authors do not forget to remind the reader that not all analyses include a high number of studies. The starting sentence "This systematic review and meta-analysis examined 33 studies…" suggests an eminent and powerful analysis, which is not exactly like that. On the contrary: the multiplicity of analysis is bringing down the strength of the conclusions, which is another argument to increase caution in the interpretation.

Although 33 studies were included in the review overall, not all contributed data to every meta-analysis, and this has now been more clearly signposted to the reader. We report the number of studies contributing to each pooled estimate in the Results section. In addition, we have revised the *Summary of Main Findings* section in the Discussion to explicitly restate the number of studies contributing to each outcome (see pg. 16).

6. The Summary includes increases in odds and risks for the different pathologies. I miss some measure of uncertainty, even if there is reasonable doubt about the accuracy of these results. I am also missing a brief reference to the detected heterogeneity in almost each analysis. The topic is commented along the document, regarding different definitions of exposure, covariates, timings and other conditions that could introduce further variability, but may be worth to recall it again in this Summary.

We have added I^2 values to all relevant tables in both the manuscript and supplementary material. To make the degree of heterogeneity and uncertainty in the pooled findings more explicit, we have revised the *Summary of Main Findings* section to emphasise that the effect estimates are subject to between-study variability (see pg. 16).

7. By the way, the point estimates don't match when I compare them. For instance, "PTB (39% increased odds or 36% increased risk)", but Figure 3 shows cOR 1.48 and cRR 1.39. And so on with LBW, SGA and NNU. Perhaps this should be clarified.

The percentages reported in the *Summary of main findings* were not updated following the updated literature search. These values have now been corrected, and we have also carefully checked all other reported point estimates throughout the manuscript to ensure consistency with the updated analyses.

8. The authors say that the countries included were the so-called “high-income” by the World Bank, but this set of countries is slightly different every year so the paper should indicate the year of reference. For instance, Russia has been excluded in the search although it was classified as “high-income country” in 2024. The update of the search could had taken this and maybe other changes into account.

We have now specified the year of the World Bank income classification used to define high-income countries in the Methods section (see pg.4).

9. Another confusion arises from this note at foot of Table 1: “Bulik et al. 2009 and Watson et al. 2017 both used the Norwegian Mother and Child Cohort Study (MoBa), therefore only the larger and more recent study (Watson et al. 2017) was included in meta-analyses to avoid double counting participants”. However, we can clearly see the study from Bulik et al at different meta-analyses: Figure 2 (cRR); Figure 3 (cRR); Figure 5 (cRR), but the name of Watson is appearing nowhere. Please clarify.

The editor has also highlighted this point, which we have addressed in comment 5 above.

10. The assessment of the risk of bias is a key topic in any systematic review. However, the instruments employed here, initially suitable for observational studies, are substantially weak, when compared with similar instruments applied to randomized controlled trials. Table 2 shows the assessments obtained with the NOS and JBI tools. Leaving aside the difficulty to answer questions like the representativeness of the cohort, or why some topics are always answered with “Yes”, I see that this method has a serious drawback: it may give the impression that a study with many positive answers is free of bias or even is as good as a RCT. Fortunately, the assessment is only used to “validate” the analysis with a subgroup of “high-quality” studies.

We agree that tools such as the Newcastle–Ottawa Scale (NOS) and the Joanna Briggs Institute (JBI) checklists are not directly comparable to instruments developed for RCTs and should not be interpreted as indicating absence of bias or equivalence to RCTs. These tools were selected because they are widely used and recommended for assessing observational studies. They were applied for descriptive purposes and not to imply a hierarchy of evidence. Studies classified as “high quality” were not assumed to be free of bias. Rather, the classifications were used to examine whether the results changed when analyses were restricted to higher-quality studies and to help indicate how confident we can be in the findings. To clarify this, we have relabeled these studies as “higher quality” and specified the number of domains in which they were rated as low risk of bias (pg. 5).

11. The analysis of sensitivity performed is not conclusive, which is actually positive, as it does not reveal strong discrepancies. Nonetheless, the authors’ interpretation feels somewhat subjective. They appear satisfied because the results from “high quality” studies *remained similar* to the whole set. However, when the type of mental health problem is taken into account, they identify changes worth noting —although such

claims remain vague due to the wide confidence intervals. Maybe, a chart would more useful for this purpose instead of a table, and the interpretation would appear objectively, with not as many words.

We agree that the sensitivity analyses did not reveal strong discrepancies, which supports the overall robustness of the findings. We also acknowledge that interpretation of subgroup analyses by type of mental health problem is limited by wide confidence intervals and the small number of studies contributing to some analyses.

While we considered presenting these results graphically, the small number of studies within subgroups and the variability between them would have limited the clarity and interpretability of additional figures. In response to the reviewer's suggestion, we have revised the text to more clearly emphasise the exploratory nature of these subgroup analyses (see pg. 15, Sensitivity Analyses section).

REVIEWER 3:

1. Overall: The exposure "pre-existing mental health problems" is not clearly defined. It is unclear whether these conditions refers to active illness or history, and how very different disorders are combined into one group, contributing to heterogeneity and difficulty in interpreting the findings. Throughout the paper, findings are frequently compared with evidence on antenatal or perinatal mental health and pregnancy outcomes. It is important to recognise that, although pre-existing mental health conditions may be associated with mental health during pregnancy, these represent distinct exposures and therefore different research questions that should be clearly separated in interpretation.

2. Abstract:
    a. What mental health problems prior to pregnancy, please clarify
    We have clarified in the abstract that the exposure of interest was any clinically diagnosed or self-reported mental health problem prior to pregnancy.

    b. What was the period of search
    We have added the search period to the abstract.

    c. Rather than repetition of exposure and outcomes in abstract the abstract could benefit from more information on methodology, what was the design of studies included, if more than one reviewer involve in screening and data extraction, Also what checklist used for quality assessment of studies
    We have added key methodological details to the abstract, including the search period, the number of reviewers involved in screening and data extraction, and the tools used for risk-of-bias assessment.

    d. Results do not specify how many studies were identified per outcome.

For readability, we did not specify the number of studies contributing to each outcome in the abstract. However, this information is reported in the Results section of the main text. To make this more explicit, we have also revised the *Summary of main findings* section in the Discussion to remind readers of the number of studies contributing to each outcome (see pg. 16).

e. Conclusion suggest "preventing the onset or recurrence of mental health problems during the perinatal period" which is out of scope of this study

We agree with the reviewer that the original wording of the conclusion may have overstated the scope of the study. We have now revised the abstract conclusion to focus on identification and support for women with mental health problems before pregnancy to inform pre-conception and maternity care planning.

3. Strengths and limitations
    a. Contradictory claim made that exposure definition was preset as a strength and this is also mentioned as limitation, this require clarification

A key strength of the review is the use of a clear and consistent definition of the exposure at the review level, specifically, pre-existing mental health problems as distinct from antenatal mental health problems. The limitation, in contrast, relates not to the definition of the exposure, but to how this exposure was measured and ascertained within studies (e.g. diagnostic codes versus self-report). Differences in how the exposure was measured may have contributed to heterogeneity and residual confounding. We have revised the text to explicitly separate these two points and avoid any contradiction.

4. Introduction
    a. Introduction focuses heavily on mental health during pregnancy, not pre-existing mental health before pregnancy, which is the actual exposure of this review

We originally included some background information on how pre-existing mental health could affect outcomes during pregnancy. Based on the reviewer's feedback, we have reduced emphasis on antenatal depression/anxiety and antidepressant exposure and reframed these studies as context rather than the primary evidence for this review.

b. Introduction drift between pre-existing, antenatal, and perinatal mental health without clearly separating these

Please see response to comment 4a above

c. Over-reliance on literature about antenatal depression/anxiety and antidepressant exposure

Please see response to comment 4a above

d. Excessive detail on long-term consequences of PTB/LBW/SGA, which is not directly needed to justify the research question

We have condensed the discussion of long-term consequences of PTB, LBW, and SGA outcomes. However, we intentionally retained a brief discussion of these longer-term consequences to justify the selection of these outcomes and to underscore their clinical and public health relevance.

e. Limited explanation of why pre-existing mental health problems could independently affect birth outcomes

We agree with the reviewer that it is important to explain why pre-existing mental health problems may independently influence birth outcomes, therefore a detailed description was included in the discussion section (pg. 18), where we expand on biological, behavioural, and social pathways and their potential contribution to adverse birth outcomes.

f. Knowledge gap is stated broadly but not clearly demonstrated in contrast to existing reviews

We have revised the introduction to clearly demonstrate the specific knowledge gap that this review addresses, highlighting how it differs from existing reviews (pg. 3).

g. Exposure definition (pre-existing mental health) is not clearly unpacked (active vs past history, chronic vs episodic, treated vs untreated).

We unpack the exposure definition in greater detail in the Methods section to improve clarity and transparency (pg. 4).

5. Methods:
    a. The exposure definition is not clearly specified at the outset. Although substance use disorders were excluded, the scope of included mental health conditions remains unclear, Broad search terms (stress, mania, PTSD) further suggest the absence of a tightly defined exposure framework. Moreover, some studies included had relied on self-reported symptoms rather than clinical diagnoses

Broad search terms were used deliberately to maximize sensitivity, as a pilot search identified very limited literature on this topic. In line with the protocol, we included studies based on both clinically diagnosed and self-reported mental health problems to ensure that we can also include population-based evidence where diagnostic data are not consistently available.

b. A large number of studies were excluded due to unclear timing of exposure, yet several included studies report “unspecified pre-existing mental health problems.”

Studies were excluded when the *timing* of mental health problems relative to pregnancy could not be established. In contrast, studies classified as reporting “unspecified pre-existing mental health problems” were included because, in line with the protocol, the exposure of interest was *any* mental health problem established prior to pregnancy.

c. Highly heterogeneous conditions (depression symptoms, clinical diagnoses, anxiety, PTSD, schizophrenia, eating disorders, affective disorders) are synthesised under a single category without justification.

The primary objective of this review was to examine the association between *any* pre-existing mental health problem and birth outcomes, reflecting the reality that mental health problems prior to pregnancy are often heterogeneous, comorbid, and variably classified in population-based data. We acknowledge that mental health problems are heterogeneous, however, as shown in Supplementary Table S5, evidence for specific pre-existing mental health problems remains limited, with relatively few studies contributing to analyses by diagnostic group. We therefore synthesised conditions under an overarching exposure to address the primary research question and explored heterogeneity through prespecified sensitivity analyses stratified by type of mental health problem, which were interpreted cautiously.

d. Study design heterogeneity (cohort and cross-sectional studies) and substantial variation in covariate adjustment are not accounted for in synthesis decisions, despite apparent pooling of adjusted estimates.

We agree that both factors are methodologically significant and can influence the comparability and interpretability of meta-analysis results. We considered conducting a sensitivity analysis by study design (cohort vs cross-sectional) however this was not feasible because only five cross-sectional studies were included in the review (Ciesielski et al., 2015; Latendresse et al., 2015; Mongan et al., 2019; Räisänen et al., 2014; Witt et al., 2012), and these studies reported varying measures of association and birth outcomes. For example, only Witt et al. (2012) and Mongan et al. (2019) reported the same outcome (LBW). Therefore, a separate meta-analysis restricted to cross-sectional studies would have included only two studies, which we considered insufficient to produce stable or meaningful pooled estimates.

We also considered conducting sensitivity analyses based on number or type of confounders. However, this was challenging due to substantial variation in the type and combination of covariates included across studies (see Table S2, Supplementary Material). Studies differed not only in the number of variables adjusted for, but also in the conceptual grouping of confounders (e.g., sociodemographic, obstetric, behavioural, and comorbidity-related factors), making it difficult to apply a consistent threshold for comparison.

e. The rationale for excluding antenatal and postnatal mental health outcomes is not explained

Antenatal mental health outcomes were excluded because there is already a substantial body of evidence examining associations between antenatal mental health problems and birth outcomes, whereas the impact of pre-existing mental health problems has not been examined systematically. Postnatal mental health outcomes were excluded due to temporality, as the outcomes of interest in this review were birth outcomes. We focused specifically on pre-existing mental health problems to avoid potential misclassification with mental health problems that have their onset during pregnancy. To emphasise this point, we have added a sentence in the introduction section (pg. 3).

f. Discrepancies in the PRISMA flow diagram require verification.

We have re-verified the study selection numbers and have uploaded a revised PRISMA flow diagram generated directly from Covidence to ensure accuracy and consistency.

6. Results
   a. Grouping exposures into clinically meaningful categories and presenting as main analysis with effect estimates would have been better

We agree that grouping exposures into clinically meaningful diagnostic categories would be valuable where sufficient data are available. However, as shown in Supplementary Table S5, the evidence for specific pre-existing mental health problems remains limited, with relatively few studies contributing to analyses within individual diagnostic groups. Conducting main analyses stratified by specific conditions would therefore have resulted in small, underpowered meta-analyses and unstable estimates. Given this, we synthesised conditions under an overarching exposure of pre-existing mental health problems to address the primary research question. To account for heterogeneity, we conducted prespecified sensitivity analyses stratified by type of mental health problem, and these findings were interpreted cautiously due to the limited number of contributing studies.

   b. Very high heterogeneity is reported but not explored or justified

We acknowledge that high statistical heterogeneity was observed across several pooled analyses. To explore potential sources of heterogeneity, we conducted prespecified sensitivity analyses stratified by: study quality, type of mental health problem, and characteristics of comparison groups. We were unable to conduct additional sensitivity analyses based on study design due to limited numbers. As demonstrated by supplementary tables S4-6, these subgroup analyses showed no clear pattern in reducing heterogeneity.

Similarly, Ghimire et al., in their meta-analysis of depression during pregnancy and adverse birth outcomes, reported I^2 values of approximately 80% for both LBW and PTB. This level of heterogeneity reflects the inherent variability in mental health research, including differences in diagnostic criteria, measurement tools, population characteristics, and adjustment for confounders.

   c. The quality assessment section should describe the specific methodological weaknesses observed across studies, rather than only reporting overall scores

We agree with the reviewer that describing methodological weaknesses across studies would be beneficial. In response, we have expanded the *Methodological quality and risk of bias* section to include a brief summary of the most common methodological weaknesses observed across studies, providing greater context beyond the overall quality classifications (see pg. 10).

7. Discussion
    a. Comparisons with antenatal mental health meta-analyses imply similar mechanisms, without acknowledging that antenatal and pre-existing conditions are epidemiologically and clinically different exposures.

We agree that pre-existing and antenatal mental health problems represent epidemiologically and clinically distinct exposures. In the Discussion section, comparisons with antenatal mental health meta-analyses were intended to provide context regarding the magnitude of associations, not to imply equivalence of exposures or mechanisms. We have added a sentence to the discussion section to emphasize this point (pg 17).

    b. The limitations suggest that differences between studies are mainly due to broad research questions and different populations, but they do not recognise that a major reason for this variation is combining very different mental health conditions into one group and pooling crude and adjusted results together.

We agree that sources of heterogeneity should be clearly acknowledged. We clarified in the limitations section that: “High heterogeneity (>75%) can be attributed to differences in populations, definitions and timing of pre-existing mental health problems, and mental health problems examined between studies.” We would also like to clarify that crude and adjusted estimates were not pooled together. They were analysed and reported separately to observe the effects of adjustment.
